# Association between bronchopulmonary dysplasia and early respiratory morbidity in children with respiratory distress syndrome: a case–control study using nationwide data

**DOI:** 10.1038/s41598-022-11657-z

**Published:** 2022-05-09

**Authors:** Jeong Eun Shin, Haerin Jang, Jung Ho Han, Joonsik Park, Soo Yeon Kim, Yoon Hee Kim, Ho Seon Eun, Soon Min Lee, Kook In Park, Myung Hyun Sohn, Min Soo Park, Kyung Won Kim

**Affiliations:** Department of Pediatrics, Yonsei University College of Medicine, 50-1 Yonsei-ro, Seodaemun-gu, Seoul, 03722 Korea

**Keywords:** Epidemiology, Respiratory distress syndrome, Risk factors

## Abstract

Bronchopulmonary dysplasia (BPD) can cause respiratory morbidity beyond the neonatal period. We aimed to analyze the association of BPD on childhood lower respiratory illness (LRI) and asthma among patients diagnosed with respiratory distress syndrome (RDS). This case–control study analyzed data between 2002 and 2015 from a nationwide database. We included 55,066 children with RDS. Two-year LRI and asthma at ages 3 and 5 were assessed. Readmission for LRIs within 2 years of birth occurred in 53.9% and 37.9% of the BPD (n = 9470) and non-BPD (n = 45,596) cases, respectively. In the BPD group, the median number of hospitalizations, mechanical ventilation and oxygen use rates were significantly higher, while the hospitalization duration was significantly longer (*P* < 0.001 for all). The relative risk of BPD was 1.42 (1.39–1.45) on total readmission and 6.53 (5.96–7.15) on intensive care unit readmission. Asthma prevalence was significantly higher in BPD group (57.6% vs. 48.9% at age 3 and 44.3% vs. 38.2% at age 5, *P* < 0.001). In children with RDS, BPD could affect repetitive and worse LRI as an independent risk factor for respiratory morbidity during the first 2 years of life. BPD may also be a crucial risk factor for asthma in preschoolers.

## Introduction

Respiratory distress syndrome (RDS) requiring surfactant replacement occurs in approximately 1% of total live births globally. RDS has been steadily increasing in Korea and in western countries^1,2^. Following the introduction of pulmonary surfactant administration in the 1990s, very low birth weight (VLBW) and extremely low birth weight (ELBW) infant survival rates in Korea increased by 1.6 and 2.7 times, respectively, over 20 years^3^.

The prevalence of bronchopulmonary dysplasia (BPD), a chronic lung disease caused by RDS, has increased because of improved preterm infant survival^4^. Despite advances in perinatal care, approximately 20–30% of VLBW infants develop BPD^1,5^. Children with BPD have higher rehospitalization rates in early childhood, mostly because of respiratory illness, longer hospital days, and higher wheezing or asthma rates than preterm or ELBW infants without BPD^6^. In Asia, few studies have investigated respiratory morbidity of BPD patients in preterms^7^. No study has evaluated the impact of BPD in children born with RDS worldwide. To our knowledge, this is the first large-scale nationwide study analyzing BPD as a risk factor for early respiratory morbidities and pediatric asthma and the first to investigate infants with RDS, not preterm or ELBW infants.

The National Health Insurance Service (NHIS) provides health insurance to most Koreans (99.01%). The NHIS covers many medical services, including medical visits, admissions, intensive care unit (ICU) care, emergency care, and pharmaceutical services. At each doctor visit, institutions submit electronic health care utilization information to the NHIS for reimbursement, which is integrated into the NHIS database^8^. The NHIS provides access to the database for research, allowing nationwide large-scale and longitudinal analyses in Korea.

We aimed to determine the influence of BPD on childhood lower respiratory illness (LRI) and asthma among a population who received pulmonary surfactant for neonatal RDS.

## Methods

### Data source and study population

All diagnosed diseases are recorded in the NHIS using codes based on the 10th Revision of the International Classification for Disease (ICD-10). From the NHIS database, we extracted data on children born between 2002 and 2015 who fulfilled either of the following criteria within 2 years of birth: diagnosed with RDS using ICD-10 code P22.0 or received exempted health insurance fees because of RDS with code V142. Patients were excluded from the study if death occurred during the initial neonatal ICU (NICU) hospitalization or if major congenital anomalies were present. Major congenital anomalies were defined as malformation or deformation of the nervous, circulatory, respiratory, digestive, or musculoskeletal systems; chromosomal abnormality; inborn errors of metabolism; or hydrops fetalis. Patients with external minor congenital anomalies, anomalies limited to the limbs, or isolated urogenital anomalies were included. ICD-10 codes used for the exclusion criteria are presented in Supplementary Table S1. Because of the nature of the data, medical records are only registered after birth registration. Hence, medical records of infants who received health care before birth registration are recorded under their birth mothers. As there were a few cases where birth registration was performed after discharge from the NICU, we excluded patients whose first medical record was registered in an outpatient clinic.

### Characteristics of RDS patients

Demographic data, diagnosed illnesses, prescribed therapies, and admission history were collected. BPD was defined as a diagnosis with ICD-10 code P27.1 within 2 years of birth. VLBW infants born after 2011 could be identified using diagnosis codes that specify birth weight < 1500 g (Supplementary Table S2). The date of the first medical record was used as the date of birth for each patient; age was calculated using this date. Comorbidity was evaluated in medical records within 2 years of birth. Patent ductus arteriosus ligation, intraventricular hemorrhage, pulmonary hypertension, necrotizing enterocolitis, retinopathy of prematurity, and neonatal sepsis were confirmed using their corresponding ICD-10 codes (Supplementary Table S3).

### Lower respiratory morbidity and mortality assessment

We evaluated medical records of patients hospitalized within 2 years of birth with a diagnosed LRI. Supplementary Table S4 contains the ICD-10 codes for bronchitis, bronchiolitis, pneumonia, croup, laryngitis, and tracheitis. The first admissions recorded for each patient were most likely NICU admissions immediately after birth; thus, we only included records after discharge from the first admission. We analyzed the number of admissions and total days admitted for both general admission and ICU care. Prescription and treatment records were used to analyze whether respiratory support was necessary. Mechanical ventilation and non-invasive ventilation were evaluated, along with oxygen prescription records. Treatment codes used to identify each treatment are listed in Supplementary Table S5.

Mortality was analyzed in all patients who died before age 3. Cause of death data was collected from the Statistics Korea database, which is based on death certificate forms that specify the primary cause of death using ICD-10 codes. We classified the cause of death into four categories: death due to respiratory diseases; death due to non-respiratory diseases; death due to external causes, including all accidents; and death due to miscellaneous causes. ICD-10 codes used for classification are organized in Supplementary Table S6.

### Asthma prevalence and morbidity assessment

Each patient was evaluated for asthma at ages 3 and 5. Asthma patients were defined as children who visited a hospital at least twice yearly with an asthma diagnosis using code J45 (asthma) or J46 (status asthmaticus), including all subsections, and prescribed with asthma medication at the same visit^9^. Asthma medications were determined based on medical charge codes for prescribed drugs (Supplementary Table S7) including oral/intravenous corticosteroids, inhaled corticosteroids, inhaled corticosteroids/long-acting beta 2 agonist combination inhalers, leukotriene receptor antagonists, short-acting beta 2 agonists for inhalation, and aminophylline/theophylline. For asthma at age 3, medical records 24–36 months after birth were retrieved, and the number of doctor visits with asthma diagnosis and asthma medication prescriptions were counted. For asthma at age 5, medical records 48–60 months after birth were retrieved to define asthma. Patients who died before reaching 24 or 48 months of age were excluded. Among asthma patients, we also examined those who were hospitalized within this period because of asthma. The number of admissions and total days admitted were used to evaluate asthma severity.

### Statistical analysis

Data were analyzed using R version 3.3 and SAS version 9.4 (SAS Institute Inc., Cary, NC). Descriptive statistics were used to summarize the entire study population. Results are presented as frequencies or medians and interquartile ranges, as appropriate. Groups were compared using Welch’s two-sample *t* test for continuous variables and Pearson’s Chi-square test with Yates’ continuity correction for categorical variables. The Mann–Whitney *U* test was used for non-normal distributions. *P* < 0.05 was considered statistically significant.

### Ethical approval

This study protocol was reviewed and approved by the Institutional Review Board of Severance Hospital (approval No. 4-2019-0383). As this was an observational study without intervention and the presentation of any identifying data, the requirement for informed consent was waived by the same IRB committee. All methods were carried out in accordance with Korean government’s guideline for health and medical data utilization.

## Results

### Respiratory morbidities/mortality within 2 years in BPD patients

From 2002 to 2015, 69,245 patients (0.95% of total live births in Korea) were diagnosed with RDS (P22.0). After applying the exclusion criteria, 55,066 patients (79.5%) were finally included in the analysis (Supplementary Fig. S1), 9470 (17.2%) of whom were diagnosed with BPD. Perinatal co-morbidities including surgically treated patent ductus arteriosus, intraventricular hemorrhage, pulmonary hypertension, necrotizing enterocolitis, retinopathy of prematurity, and neonatal sepsis were significantly higher in the BPD group than the non-BPD group (*P* < 0.001, Table 1). Rehospitalization for LRI within 2 years of birth occurred in 53.9% of BPD patients and 37.9% of non-BPD patients (*P* < 0.001, Table 1).

**Table 1:** Patient characteristics.

	BPD (n = 9470)	Non-BPD (n = 45,596)	*P*-value
Male	5176 (54.7)	26,321 (57.7)	< 0.001
**Perinatal comorbidity**			
PDA, treated surgically	184 (1.9)	91 (0.2)	< 0.001
Intraventricular hemorrhage	528 (5.6)	287 (0.6)	< 0.001
Pulmonary hypertension	824 (8.7)	1266 (2.8)	< 0.001
Necrotizing enterocolitis	1018 (10.7)	941 (2.1)	< 0.001
Retinopathy of prematurity	7460 (78.8)	17,274 (37.9)	< 0.001
Neonatal sepsis	4849 (51.2)	11,261 (24.7)	< 0.001
Readmission for LRI within 2 years of birth	5102 (53.9)	17,273 (37.9)	< 0.001
Death within 2 years of birth	264 (2.8)	387 (0.8)	< 0.001

Data are expressed as n (%).

*LRI* lower respiratory illness, *BPD* bronchopulmonary dysplasia, *PDA* patent ductus arteriosus.

*P*-value was calculated using Welch’s two-sample *t* test for continuous variables and Pearson’s Chi-square test with Yates’ continuity correction for categorical variables.

Among readmitted patients, the median number of readmissions within 2 years of birth was twice as high in the BPD group than the non-BPD group (2 (1–40) vs. 1 (1–29), *P* < 0.001, Table 2). Patients in the BPD group showed a significantly longer hospital stay and higher rates of mechanical ventilator, non-invasive ventilator, and oxygen use than those in the non-BPD group (14.9% vs. 2.7%, 3.9% vs. 0.5%, and 41.0% vs. 14.5%, respectively, *P* < 0.001, Table 2). Furthermore, compared with those in the non-BPD group, patients in the BPD group were 4.7 times more likely to require ICU care and stayed in the ICU 2.4 times longer (*P* < 0.001, Table 2).

**Table 2:** Patients readmitted within 2 years of birth because of lower respiratory illness.

	BPD (n = 5102)	Non-BPD (n = 17,273)	*P*-value
Number of readmissions*	2 (1–40)	1 (1–29)	< 0.001
Hospital stay, days	13 (6–30)	8 (5–15)	< 0.001
Ventilator use	760 (14.9)	459 (2.7)	< 0.001
NIV use	201 (3.9)	82 (0.5)	< 0.001
Oxygen use	2094 (41.0)	2505 (14.5)	< 0.001
ICU admission	1030 (20.1)	760 (4.3)	< 0.001
Number of ICU admissions*	1 (1–6)	1 (1–8)	< 0.001
ICU hospital stay, days	34 (18–61)	14 (9–25)	< 0.001

Data are expressed as n (%), median (range)*, or median (interquartile range). The Mann–Whitney *U* test was used for non-normal distributions in continuous variables.

*BPD* bronchopulmonary dysplasia, *NIV* non-invasive ventilator, *ICU* intensive care unit.

**Figure 1:**
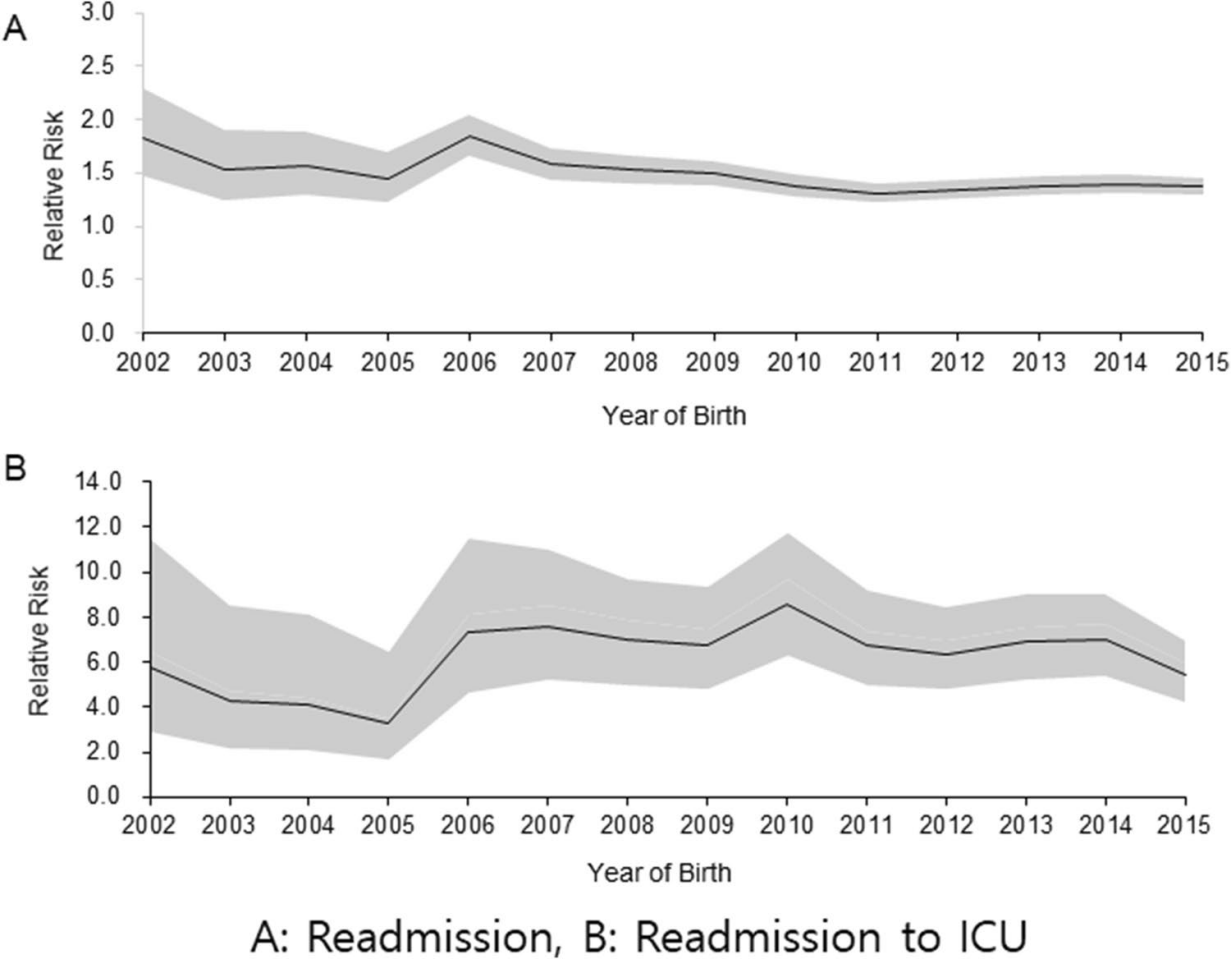
Trend in the relative risk of bronchopulmonary dysplasia on total readmission (**A**) and ICU admission (**B**) because of respiratory diseases. *ICU* intensive care unit.

The relative risk (RR) of BPD on readmission because of LRI significantly and steadily increased from 2002 to 2015 (1.42, 95% confidence interval [CI], 1.39–1.45, Fig. 1A). The RR of BPD on ICU admission was much higher than that on total readmission (6.53, 95% CI, 5.96–7.15, Fig. 1B). The RRs of both total readmission and ICU admission showed an increasing trend between 2005 and 2006; the RR of ICU admission peaked in 2010 (8.60, 95% CI, 6.32–11.70, Fig. 1).

Of the 55,066 patients, 651 (1.18%) died within 2 years. The mortality rate was significantly higher in the BPD group than the non-BPD group (2.8% vs. 0.8%, *P* < 0.001, Table 1). Respiratory morbidity caused 42.4% and 25.3% of deaths in the BPD and non-BPD groups, respectively (*P* < 0.001, Supplementary Table S8).

### Respiratory morbidities in VLBW infants

Of the total population, 33,437 patients (60.7%) were included in a subgroup analysis based on birth weight of < 1500 g or > 1500 g. BPD was diagnosed in 58.7% and 7.2% of the < 1500 g and > 1500 g groups, respectively. Compared with the non-BPD group, the BPD group had a significantly higher readmission frequency; more days in the hospital; and higher mechanical ventilator, non-invasive ventilator, and oxygen use on readmission within 2 years in both the < 1500 g and > 1500 g birth weight subgroups. The number of ICU admissions was not higher in patients with BPD in the > 1500 g group (Supplementary Table S9).

Patients with BPD showed similar RRs for readmission because of LRI in both the < 1500 g (1.23, 95% CI 1.17–1.29, Supplementary Fig. S2) and > 1500 g groups (1.28, 95% CI 1.22–1.34). All patients with RDS born between 2011 and 2015 had an RR for readmission of 1.36 (Supplementary Fig. S2).

### Asthma prevalence and asthma-related morbidities in BPD

**Figure 2:**
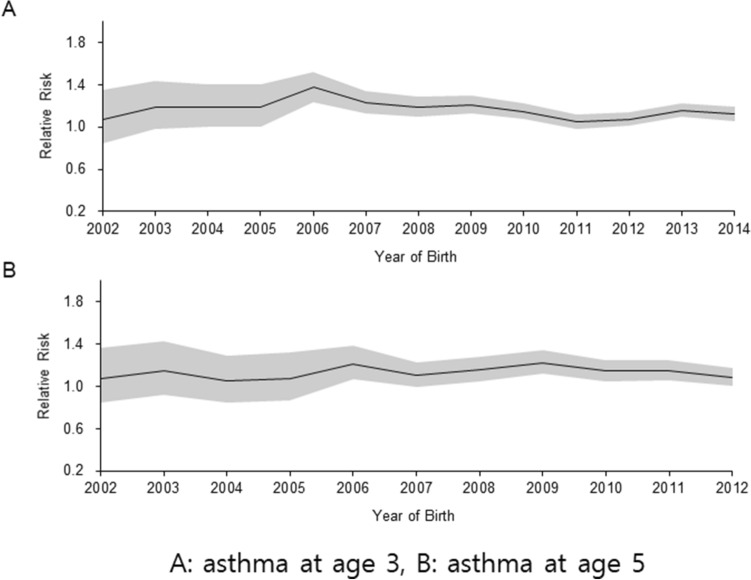
Relative risk of bronchopulmonary dysplasia on asthma diagnosis at ages 3 (**A**) and 5 (**B**).

Patients born between 2002 and 2012 were included in the analysis. Of 33,129 children, asthma prevalence at ages 3 and 5 was 49.7% and 38.7%, respectively. The proportion of patients diagnosed with asthma was significantly higher in the BPD group than the non-BPD group at both ages 3 (57.6% vs. 48.9%, *P* < 0.001) and 5 (44.3% vs. 38.2%, *P* < 0.001). Total asthma prevalence decreased at age 5 (Table 3). The number of hospital admissions for asthma and the duration of hospitalization among admitted patients were higher in the BPD group than in the non-BPD group (Table 3). The RR of BPD on asthma diagnosis was 1.06–1.37 at age 3 and 1.05–1.23 at age 5 (Fig. 2).

**Table 3:** Asthma patients categorized per BPD status.

	BPD (n = 5760)	Non-BPD (n = 27,369)	*P*-value
**Asthma at age 3 (n = 16,467)**	3217 (57.6)	13,250 (48.9)	< 0.001
Number of admitted patients^a^	772 (24.0)	2289 (17.3)	< 0.001
Number of admissions per person*	1 (1–13)	1 (1–12)	0.157
Hospital days per person	7 (5–12)	6 (5–10)	0.003
**Asthma at age 5 (n = 12,812)**	2468 (44.3)	10,344 (38.2)	< 0.001
Admissions due to asthma^b^	369 (15.0)	1070 (10.3)	0.439
Number of admissions per person*	1 (1–12)	1 (1–11)	< 0.001
Hospital days per person	6 (4–10)	6 (5–9)	0.777

Data are expressed as n (%), median (interquartile range), or median (range)*.

^a^Between 36 and 48 months of age.

^b^Between 60 and 72 months of age.

The percentage of patients with asthma at ages 3 and 5 was calculated using the total number of patients alive at ages 3 and 5.

*BPD* bronchopulmonary dysplasia.

## Discussion

This study analyzed the association between BPD and childhood respiratory morbidities in Korean children with RDS born between 2002 and 2015. In the study population, 17.2% of children were diagnosed with BPD; 58.7% of the < 1500 g and 7.2% of the > 1500 g birth weight subgroups had BPD. The incidence and severity of LRIs requiring readmission within 2 years of birth were significantly higher in the BPD group than the non-BPD group. BPD increased the frequency of readmission, hospitalization stay, ICU admission rate and duration, and overall ventilator and oxygen use in admitted patients. The RR of BPD on rehospitalization slightly decreased after adjusting for birth weight; however, BPD was still significantly associated with rehospitalization in both birth weight groups. Pediatric asthma prevalence and severity were also higher in the BPD group than the non-BPD group at ages 3 and age 5, approximately 3 times higher than in the general population.

The high incidence of early life LRIs in BPD patients means they are vulnerable to respiratory infections and potential further decline of lung function because of LRI-related causes. BPD is characterized by disrupted alveolar and vascular growth due to pulmonary inflammation, oxidative stress, and mechanical trauma to the fragile immature lungs^10^. Most alveolarization occurs within the first 2 years of life, and pulmonary function follows a predictable progression from birth through childhood until full maturation at age 22; therefore, any adverse exposure before or during development has the potential to change the trajectory of lung development and cause a loss of lung function with age^10–13^. Also, even late preterm children had decreased childhood lung function compared with those delivered at term^14^.

The RR of BPD on readmission decreased from 2002 to 2005 but rapidly increased during 2005–2006, which was further emphasized when looking at ICU admission (Fig. 1). This result can be partially explained by the increase in preterm infant survival. In VLBW infants, the survival rate of infants born in Korea between 2005 and 2009 was 85.3%, up 14.5% from that of infants born between 2000 and 2004 (74.5%)^15^. When comparing 2000 and 2010, preterm births increased both in absolute number (23,914 vs. 27,304) and in the proportion of total births (3.8% vs 5.8%), although the total number of births decreased by 35%^16^. The proportion of BPD in the RDS population increased to 23.1% in 2007, compared with 17.0% in 2002^16^. Two epidemics of mycoplasma pneumonia occurred, in 2006 and from 2010 to 2011^17^, which might have affected BPD patients more severely. Rehospitalization rates in children with BPD during the first 2 years of life were similar to those reported in the United States^18,19^.

A recent study by Sol et al.^9^ showed that the average prevalence of asthma in all Korean children born between 2002 and 2015 peaked at 18.4% at age 3 and decreased to 14.7% at age 5. Therefore, we can infer that the prevalence of asthma is approximately 3 times higher in BPD patients than in the general population. Moreover, non-BPD patients with RDS were diagnosed with asthma 2.5 times more than the general population. Further investigation using a large-scale longitudinal cohort study containing detailed medical records is necessary to clarify the predominant risk factors for this high prevalence of asthma in the RDS population.

Interestingly, the prevalence of asthma was not affected by BPD when we limited the population to only < 1500 g birth weight infants (data not shown); however, LRI-related readmission was influenced by BPD in the < 1500 g birth weight subgroup. This finding is partially consistent with the results of Jackson et al.^20^ who suggested asthma diagnosis in children born before 28 weeks gestation is predominantly associated with post-NICU risk factors, especially low socioeconomic status, but not with neonatal complications, including BPD. Kim et al.^21^ reported that small for gestational age infants were associated with poor pulmonary function showing obstructive patterns within BPD patients within the first year of life. Therefore, the influence of BPD on asthma may be diminished when the population is limited to extremely preterm or VLBW infants because birth weight and prematurity are relatively predominant risk factors for asthma. Moreover, because asthma development is affected by multiple factors including genetic, prenatal, prematurity, birth weight, and postnatal environmental factors^22,23^, the impact of BPD on asthma may be less than that on LRIs. We found asthma-related hospitalization and the influence of BPD on asthma decreased at age 5 than at age 3, suggesting the influence of BPD decreases as children age. However, asthma development in BPD patients can result from LRI-related hospitalizations and their severity during the first 2 years of life. Previous studies have shown asthma is closely related to early life respiratory syncytial virus infection, and viral prophylaxis reduced childhood asthma prevalence^23–25^.

BPD diagnosis in early life can cause an early decline in adult lung function. Several longitudinal cohort studies revealed a decreased forced expiratory volume and decreased forced expiratory volume/forced vital capacity ratio at school age in preterm-born children^26,27^. Decreased lung function in childhood has also been suggested to be a risk factor for adult chronic obstructive pulmonary disorder^28–30^. BPD patients may persistently present with obstructive lung disease into adulthood^31,32^. However, long-term outcomes of survivors with BPD who were born before the current era of perinatal care may not be generalizable to more recent survivors. Long-term follow-up is necessary to investigate whether prematurity of BPD itself is a predominant risk factor for respiratory morbidities or if BPD is only a major synergistic factor.

Previous studies analyzed BPD as a risk factor for respiratory morbidity or asthma in VLBW or extremely preterm infants. Our study focused on a nationwide population with RDS requiring surfactant replacement. As the diagnostic code in claims data depends on input by each physician, the accuracy of diagnostic code input is critical. The diagnostic code for “RDS” must be accompanied by a pulmonary surfactant prescription and registered with the rare incurable disease code V142, which provides financial benefits for RDS patients from the national health insurance. Extraction of patient data using code V142 is more accurate and reliable than the diagnostic code related to ‘prematurity’ which has not been regulated by obligation for prescription or redemptions. We were able to compare the asthma prevalence in the RDS group with that in the general population using the same operational diagnosis^9^. However, as this is large-scale real-world data, the possibility of prescribing asthma medications for preventive purposes cannot be excluded in some BPD patients.

This study has some limitations, including those inherent in any retrospective study using claims data. Although a large number of participants in our study allowed us to investigate patients with RDS extensively, we could not obtain all perinatal and environmental variables because of insufficient detailed medical records. Therefore, we were unable to distinguish the extent of immaturity and BPD on respiratory morbidity. BPD diagnosis in our study population was based on the input of a diagnostic code by physicians and was presumed to be defined as “oxygen use for 28 days”. Subgroup analysis among VLBW infants was only possible in infants born after 2011 when the Korean Classification of Disease was updated. Infants with transient tachypnea of newborn (TTN) or pneumonia may have been incorrectly diagnosed as RDS and included in our study population. However, previous national multicenter studies reported that 93.5% and 87.9% of total RDS patients were < 37 weeks and < 2500 g respectively, whereas patients with TTN or pneumonia constituted only minimal portion in preterm or low birth weight infants^33,34^. Therefore, we suggest that the overlapping or misdiagnosis of TTN or pneumonia with RDS in our cohort has minimal effects on the conclusion we draw from the study.

## Conclusion

By investigating the annual trends of decreased BPD mortality for over 10 years, we were able to explore the association between BPD and respiratory morbidity from a macroscopic viewpoint. In addition, we revealed the real burden on readmitted patients by focusing on the severity of the patients.

Our study demonstrated that children with BPD required rehospitalization because of respiratory morbidities twice as frequently as children without BPD. The BPD population received approximately five times more intensive care, assessed by ventilator use and ICU admission rate than the non-BPD population. The prevalence of pediatric asthma at ages 3 and 5 were three times higher in BPD children than in the general population. Regular follow-up and care for patients with BPD are crucial, and a follow-up plan may be necessary to manage their long-term health.

## Supplementary Information

Supplementary Information.

## Data Availability

The data that support the findings of this study are available from the National Health Insurance Service (Wonju-si, South Korea) but restrictions apply to the availability of these data, which were used under license for the current study, and so are not publicly available. Data are however available from the National Health Insurance Service upon reasonable request using identical data extraction methods stated in our methods and used within the service regulations.
