# Author Correction: Association between bronchopulmonary dysplasia and early respiratory morbidity in children with respiratory distress syndrome: a case–control study using nationwide data

**DOI:** 10.1038/s41598-022-13939-y

**Published:** 2022-06-01

**Authors:** Jeong Eun Shin, Haerin Jang, Jung Ho Han, Joonsik Park, Soo Yeon Kim, Yoon Hee Kim, Ho Seon Eun, Soon Min Lee, Kook In Park, Myung Hyun Sohn, Min Soo Park, Kyung Won Kim

**Affiliations:** Department of Pediatrics, Yonsei University College of Medicine, 50-1 Yonsei-ro, Seodaemun-gu, Seoul, 03722 Korea

Correction to: *Scientific Reports* 10.1038/s41598-022-11657-z, published online 09 May 2022

The original version of this Article contained an error in the Acknowledgments section.

"This study was supported by a Severance Hospital Research fund for Clinical excellence (SHRC) (C-2020–0014) and a grant (MFDS2022–178) from Ministry of Food and Drug Safety in 2022."

now reads:

"This study was supported by a Severance Hospital Research fund for Clinical excellence (SHRC) (C-2020–0014) and a grant (22213MFDS486) from Ministry of Food and Drug Safety in 2022."

The original Article has been corrected.

