# Supplementary material for: Association between bronchopulmonary dysplasia and early respiratory morbidity in children with respiratory distress syndrome: a case–control study using nationwide data
Source: Sci Rep. 2022 May 9;12:7578. doi: 10.1038/s41598-022-11657-z (PMC9085740; doi:10.1038/s41598-022-11657-z)
Supplement: Supplementary file 1 — Supplementary Information. [file 41598_2022_11657_MOESM1_ESM.docx]

**Supplementary Information**

**Supplementary Table S1.** ICD-10 codes used to identify congenital anomalies for the exclusion criteria

**Supplementary Table S2.** ICD-10 codes used to specify birth weight of under 1500g

**Supplementary Table S3.** ICD-10 codes used to identify co-morbid diseases

**Supplementary Table S4.** ICD-10 codes used to identify lower respiratory diseases

**Supplementary Table S5.** Treatment codes used to identify ventilator use, non-invasive ventilator use, and oxygen prescription during hospital admissions

**Supplementary Table S6.** Classification of cause of death using ICD-10 codes

**Supplementary Table S7.** Active ingredient codes used to identify asthma medications prescribed for asthma patients

**Supplementary Table S8.** Cause of death among patients who died within 2 years of birth

**Supplementary Table S9.** Readmission due to lower respiratory disease in 2 years (2011-2015)

**Supplementary Figure S1.** Patient flow diagram and analysis subjects

**Supplementary Figure S2.** Relative risk of BPD on readmission due to respiratory diseases

**Supplementary Table S1. ICD-10 codes used to identify congenital anomalies for the exclusion criteria**

| **ICD-10 Code,  with subsections** | **Term** |
| --- | --- |
| P56.0 | Hydrops fetalis due to isoimmunization |
| P56.9 | Hydrops fetalis due to other and unspecified hemolytic disease |
| P83.2 | Hydrops fetalis not due to hemolytic disease |
| E70 | Disorders of aromatic amino-acid metabolism |
| E70.0 | Classical phenylketonuria |
| E70.1 | Other hyperphenylalaninemias |
| E70.2 | Disorders of tyrosine metabolism |
| E70.2 | Alkaptonuria |
| E70.2 | Hyperytyrosinemia |
| E70.2 | Ochronosis |
| E70.2 | Tyrosinaemia |
| E70.2 | Tyrosinosis |
| E70.3 | Albinism |
| E70.3 | Oculocutaneous albinism |
| E70.3 | Ocular albinism |
| E70.3 | Chediack(-Steinbrinck)-Higashi syndrome |
| E70.3 | Cross syndrome |
| E70.3 | Hermansky-Pudlak syndrome |
| E70.8 | Other disorders of aromatic amino-acid metabolism |
| E70.8 | Disorder of histidine metabolism |
| E70.8 | Disorder of tryptophan metabolism |
| E70.9 | Disorder of aromatic amino-acid metabolism, unspecified |
| E71 | Disorders of branched-chain amino-acid metabolism and fatty-acid metabolism |
| E71.0 | Maple-syrup-urine disease |
| E71.1 | Other disorders of branched-chain amino-acid metabolism |
| E71.1 | Hyperleucine-isoleucinemia |
| E71.1 | Hypervalinemia |
| E71.1 | Methylmalonic acidemia |
| E71.1 | Isovaleric acidemia |
| E71.1 | Propionic acidemia |
| E71.2 | Disorder of branched-chain amino-acid metabolism, unspecified |
| E71.3 | Disorders of fatty-acid metabolism |
| E71.3 | Adrenoleukodystrophy[Addison-Schilder] |
| E71.3 | Muscle carnitine palmityltransferase deficiency |
| E72 | Other disorders of amino-acid metabolism |
| E72.0 | Disorders of amino-acid transport |
| E72.0 | Cystine storage disease(N29.8*) |
| E72.0 | Cystinosis |
| E72.0 | Cystinuria |
| E72.0 | Fanconi (-de Toni)(-Debre) syndrome |
| E72.0 | Hartnup's disease |
| E72.0 | Lowe's syndrome |
| E72.1 | Disorder of sulfur-bearing amino-acid metabolism |
| **ICD-10 Code,  with subsections** | **Term** |
| E72.1 | Cystathioninuria |
| E72.1 | Homocystinuria |
| E72.1 | Methioninaemia |
| E72.1 | Sulfite oxidase deficiency |
| E72.2 | Disorders of urea cycle metabolism |
| E72.2 | Argininemia |
| E72.2 | Argininosuccinic aciduria |
| E72.2 | Citrullinaemia |
| E72.2 | Hyperammonaemia |
| E72.3 | Disorders of lysine and hydroxylysine metabolism |
| E72.3 | Glutaric aciduria |
| E72.3 | Hydroxylysinaemia |
| E72.3 | Hyperlysinaemia |
| E72.4 | Disorders of ornithine metabolism(E72.4) |
| E72.4 | Ornithinaemia( type Ⅰ,Ⅱ) |
| E72.5 | Disorders of glycine metabolism |
| E72.5 | Hyperhydroxyprolinemia |
| E72.5 | Hyperprolinemia (type Ⅰ,Ⅱ) |
| E72.5 | Non-ketotic hyperglycinemia |
| E72.5 | Sarcosinemia |
| E72.8 | Other specified disorders of amino-acid metabolism |
| E72.8 | Disorders of β-amino-acid metabolism |
| E72.8 | Disorders of γ-glutamyl cycle |
| E72.9 | Disorder of amino-acid metabolism, unspecified |
| E74 | Other disorders of carbohydrate metabolism |
| E74.0 | Glycogen storage disease |
| E74.0 | Cardiac glycogenosis |
| E74.0 | Andersen's disease |
| E74.0 | Cori's disease |
| E74.0 | Forbes's disease |
| E74.0 | Her's disease |
| E74.0 | McArdl's disease |
| E74.0 | Pompe's disease |
| E74.0 | Tarui's disease |
| E74.0 | von Gierke's disease |
| E74.0 | Liver phosphorylase deficiency |
| E74.1 | Disorders of fructose metabolism |
| E74.1 | Essential fructosuria |
| E74.1 | Fructose-1, 6-diphosphatase deficiency |
| E74.1 | Hereditary fructose intolerance |
| E74.2 | Disorders of galactose metabolism |
| E74.2 | Galactokinase deficiency |
| E74.2 | Galactosemia |
| E74.3 | Other disorders of intestinal carbohydrate absorption |
| E74.3 | Glucose-galactose malabsorption |
| **ICD-10 Code,  with subsections** | **Term** |
| E74.3 | Sucrase deficiency |
| E74.4 | Disorders of pyruvate metabolism and gluconeogenesis |
| E74.4 | Deficiency of phosphoenol-pyruvate carboxykinase |
| E74.4 | Deficiency of carboxylase pyruvate |
| E74.4 | Deficiency of dehydrogenase pyruvate |
| E74.8 | Other specified disorders of carbohydrate metabolism |
| E74.8 | Essential pentosuria |
| E74.8 | Oxalosis |
| E74.8 | Oxaluria |
| E74.8 | Renal glycosuria |
| E74.9 | Disorder of carbohydrate metabolism, unspecified |
| E75 | Disorders of sphingolipid metabolism and other lipid storage disorders |
| E75.0 | GM₂-gangliosidosis |
| E75.0 | Sandhoff's disease |
| E75.0 | Tay-Sachs's disease |
| E75.0 | GM₂ gangliosidosis NOS |
| E75.0 | Adult GM₂ gangliosidosis |
| E75.0 | Juvenile GM₂ gangliosidosis |
| E75.1 | Other gangliosidosis |
| E75.1 | Gangliosidosis NOS |
| E75.1 | GM₁- gangliosidosis |
| E75.1 | GM₃- gangliosidosis |
| E75.1 | Mucolopidosis Ⅳ |
| E75.2 | Other sphingolipidosis |
| E75.2 | Fabry's(- Anderson ) disease |
| E75.2 | Gaucher's disease |
| E75.2 | Krabbe's disease |
| E75.2 | Niemann - Pick's disease |
| E75.2 | Farber's syndrome |
| E75.2 | Metachromatic leukodystrophy |
| E75.2 | Sulfatase deficiency |
| E75.3 | Sphingolipidosis, unspecified |
| E75.4 | Neuronal ceroid lipofuscinosis |
| E75.4 | Batten's disease |
| E75.4 | Jansky-Bielschowsky's disease |
| E75.4 | Kufs's disease |
| E75.4 | Spielmeyer-Vogt's disease |
| E75.5 | Other lipid storage disorders |
| E75.5 | Cerebrotendinous cholesterosis[van Bogaert-Scherer- Epstein] |
| E75.5 | Wolman's disease |
| E75.6 | Lipid storage disorder, unspecified |
| E76 | Disorders of glycosaminoglycan metabolism |
| E76.0 | Mucopolysaccharidosis, type I |
| E76.0 | Hurler syndrome |
| **ICD-10 Code,  with subsections** | **Term** |
| E76.0 | Hurler-Scheie syndrome |
| E76.0 | Scheie syndrome |
| E76.1 | Mucopolysaccharidosis, type II |
| E76.1 | Hunter's syndrome |
| E76.2 | Other mucopolysaccharidoses |
| E76.2 | β-Glucuronidase deficiency |
| E76.2 | Mucopolysaccharidosis,types Ⅲ,Ⅳ,Ⅵ,Ⅶ |
| E76.2 | Maroteaux-Lamy syndrome(mild, severe) |
| E76.2 | Morquio syndrome (-like, classic) |
| E76.2 | Sanfilippo (types B,C,D)syndrome |
| E76.3 | Mucopolysaccharidosis, unspecified |
| E76.8 | Other disorders of glucosaminoglycan metabolism |
| E76.9 | Disorders of glucosaminoglycan metabolism, unspecified |
| E79 | Disorders of purine and pyrimidine metabolism |
| E79.0 | Hyperuricemia without signs of inflammatory arthritis and tophaceous disease |
| E79.0 | Asymptomatic hyperuricemia |
| E79.1 | Lesch-Nyhan syndrome |
| E79.8 | Other disorders of purine and pyrimidine metabolism |
| E79.8 | Hereditary xanthinuria |
| E79.9 | Disorder of purine and pyrimidine metabolism, unspecified |
| E80 | Disorders of porphyrin and bilirubin metabolism |
| E80 | Defects of catalase and peroxidase |
| E80.0 | Hereditary erythropoietic porphyria |
| E80.0 | Congenital erythropoietic porphyria |
| E80.0 | Erythropoietic protoporphyria |
| E80.1 | Porphyria cutanea tarda |
| E80.2 | Other porphyria |
| E80.2 | Hereditary coproporphyria |
| E80.2 | Porphyria NOS |
| E80.2 | Acute intermittent (hepatic) porphyria |
| E80.3 | Defects of catalase and peroxidase |
| E80.3 | Acatalasia[Takahara] |
| E80.4 | Gilbert's syndrome |
| E80.5 | Crigler-Najjar syndrome |
| E88.9 | Metabolic disorder, unspecified |
| G71.2 | Congenital myopathies |
| G71.2 | Congenital muscular dystrophy NOS |
| G71.2 | Congenital muscular dystrophy with specific morphological abnormalities of the muscle fibre |
| G71.2 | Central core disease |
| G71.2 | Minicore disease |
| G71.2 | Multicore disease |
| G71.2 | Fibre-type disproportion |
| G71.2 | Myotubular(centronluclear) myopathy |
| G71.2 | Nemaline myopathy |
| Q00 | Anencephaly and similar malformations |
| **ICD-10 Code,  with subsections** | **Term** |
| Q00.0 | Anencephaly |
| Q00.0 | Acephaly |
| Q00.0 | Acrania |
| Q00.0 | Amyelencephaly |
| Q00.0 | Hemianencephaly |
| Q00.0 | Hemicephaly |
| Q00.1 | Craniorachischiasis |
| Q00.2 | Iniencephaly |
| Q01 | Encephalocele |
| Q01 | Encephalomyelocele |
| Q01 | Hydroencephalocele |
| Q01 | Hydromeningocele, cranial |
| Q01 | Meningocele, cerebral |
| Q01 | Meningoencephalocele |
| Q01.0 | Frontal encephalocele |
| Q01.1 | Nasofrontal encephalocele |
| Q01.2 | Occipital encephalocele |
| Q01.8 | Encephalocele of other sites |
| Q01.9 | Encephalocele, unspecified |
| Q02 | Microcephaly |
| Q02 | Hydromicrocephaly |
| Q02 | Micrencephalon |
| Q03 | Congenital hydrocephalus |
| Q03 | Hydrocephalus in newborn |
| Q03.0 | Malformations of aqueduct of Sylvius |
| Q03.0 | Aqueduct of Sylvius anomaly |
| Q03.0 | Aqueduct of Sylvius obstruction, congenital |
| Q03.0 | Aqueduct of Sylvius stenosis |
| Q03.1 | Atresia of foramina of Magendie and Luschka |
| Q03.1 | Dandy-Walker syndrome |
| Q03.8 | Other congenital hydrocephalus |
| Q03.9 | Congenital hydrocephalus, unspecified |
| Q04 | Other congenital malformations of brain |
| Q04.0 | Congenital malformation of corpus callosum |
| Q04.0 | Agenesis of corpus callosum |
| Q04.1 | Arhinencephaly |
| Q04.2 | Holoprosencephaly |
| Q04.3 | Other reduction deformities of brain |
| Q04.3 | Absence of part of brain |
| Q04.3 | Agenisis of part of brain |
| Q04.3 | Aplasia of part of brain |
| Q04.3 | Hypoplasia of part of brain |
| Q04.3 | Agyria |
| Q04.3 | Hydranencephaly |
| Q04.3 | Lissencephaly |
| Q04.3 | Microgyria |
| **ICD-10 Code,  with subsections** | **Term** |
| Q04.3 | Pachygyria |
| Q04.4 | Septo-optic dysplasia |
| Q04.5 | Megalencephaly |
| Q04.6 | Congenital cerebral cysts |
| Q04.6 | Porencephaly |
| Q04.6 | Schizencephaly |
| Q04.8 | Other specified congenital malformations of brain |
| Q04.8 | Macrogyria |
| Q04.9 | Congenital malformation of brain, unspecified |
| Q04.9 | Congenital anomaly of brain NOS |
| Q04.9 | Congenital deformity of brain NOS |
| Q04.9 | Congenital disease or lesion of brain NOS |
| Q04.9 | Congenital multiple anomalies of brain NOS |
| Q05 | Spina bifida |
| Q05 | Hydromeningocele (spinal) |
| Q05 | Meningocele(spinal) |
| Q05 | Meningomyelocele |
| Q05 | Myelocele |
| Q05 | Myelomenigocele |
| Q05 | Rachischisis |
| Q05 | Bifida spina (aperta) (cystica) |
| Q05 | Syringomyelocele |
| Q05.0 | Cervical spina bifida with hydrocephalus |
| Q05.1 | Thoracic spina bifida with hydrocephalus |
| Q05.1 | Dorsal spina bifida with hydrocephalus |
| Q05.1 | Thoracolumbar spina bifida with hydrocephalus |
| Q05.2 | Lumbar spina bifida with hydrocephalus |
| Q05.2 | Lumbosacral spina bifida with hydrocephalus |
| Q05.3 | Sacral spina bifida with hydrocephalus |
| Q05.4 | Unspecified spina bifida with hydrocephalus |
| Q05.5 | Cervical spina bifida without hydrocephalus |
| Q05.6 | Thoracic spina bifida without hydrocephalus |
| Q05.6 | Dorsal spina bifida NOS |
| Q05.6 | Thoracolumbar spina bifida NOS |
| Q05.7 | Lumbar spina bifida without hydrocephalus |
| Q05.7 | Lumbosacral spina bifida NOS |
| Q05.8 | Sacral spina bifida without hydrocephalus |
| Q05.9 | Spina bifida, unspecified |
| Q06 | Other congenital malformations of spinal cord |
| Q06.0 | Amyelia |
| Q06.1 | Hypoplasia and dysplasia of spinal cord |
| Q06.1 | Atelomyelia |
| Q06.1 | Myelatelia |
| Q06.1 | Myelodysplasia of spinal cord |
| Q06.2 | Diastematomyelia |
| Q06.3 | Other congenital cauda equina malformations |
| **ICD-10 Code,  with subsections** | **Term** |
| Q06.4 | Hydromyelia |
| Q06.4 | Hydrorachis |
| Q06.8 | Other specified congenital malformations of spinal cord |
| Q06.9 | Congenital malformation of spinal cord, unspecified |
| Q06.9 | Congenital anomaly of spinal cord or meninges NOS |
| Q06.9 | Congenital deformity of spinal cord or meninges NOS |
| Q06.9 | Congenital disease or lesion of spinal cord or meninges NOS |
| Q07 | Other congenital malformations of nervous system |
| Q07.0 | Arnold-Chiari syndrome |
| Q07.8 | Other specified congenital malformations of nervous system |
| Q07.8 | Agenisis of nerve |
| Q07.8 | Displacement of brachial plexus |
| Q07.8 | Jaw-winking syndrome |
| Q07.8 | Marcus-Gunn's syndrome |
| Q07.9 | Congenital malformation of nervous system, unspecified |
| Q07.9 | Congenital anomaly of nervous system NOS |
| Q07.9 | Congenital deformity of nervous system NOS |
| Q07.9 | Congenital disease or lesion of nervous system NOS |
| Q11 | Anophthalmos, microphthalmos and macrophthalmos |
| Q11.0 | Cystic eyeball |
| Q11.1 | Other anopthalmos |
| Q11.1 | Agenesis of eye |
| Q11.1 | Aplasia of eye |
| Q11.2 | Microphthalmos |
| Q11.2 | Cryptophthalmos NOS |
| Q11.2 | Dysplasia of eye |
| Q11.2 | Hypoplasia of eye |
| Q11.2 | Rudimentary eye |
| Q11.3 | Macrophthalmos |
| Q12 | Congenital lens malformations |
| Q12.0 | Congenital cataract |
| Q12.1 | Congenital displaced lens |
| Q12.2 | Coloboma of lens |
| Q12.3 | Congenital aphakia |
| Q12.4 | Spherophakia |
| Q12.8 | Other congenital lens malformations |
| Q12.9 | Congenital lens malformation, unspecified |
| Q13 | Congenital malformations of anterior segment of eye |
| Q13.0 | Coloboma of iris |
| Q13.0 | Coloboma NOS |
| Q13.1 | Absence of iris |
| Q13.1 | Aniridia |
| Q13.2 | Other congenital malformation of iris |
| Q13.2 | Anisocoria, congenital |
| Q13.2 | Atresia of pupil |
| Q13.2 | Congenital malformation of iris NOS |
| **ICD-10 Code,  with subsections** | **Term** |
| Q13.2 | Corectopia |
| Q13.3 | Congenital corneal opacity |
| Q13.4 | Other congenital corneal malformations |
| Q13.4 | Congenital malformation of cornea NOS |
| Q13.4 | Microcornea |
| Q13.4 | Peter's anomaly |
| Q13.5 | Bule sclera |
| Q13.8 | Other congenital malformations of anterior segment of eye |
| Q13.8 | Rieger's anomaly |
| Q13.9 | Congenital malformation of anterior segment of eye, unspecified |
| Q14 | Congenital malformations of posterior segment of eye |
| Q14.0 | Congenital malformation of vitreous humour |
| Q14.0 | Congenital vitreous opacity |
| Q14.1 | Congenital malformation of retina |
| Q14.1 | Congenital retinal aneurysm |
| Q14.2 | Congenital malformation of optic disc |
| Q14.2 | Coloboma of optic disc |
| Q14.3 | Congenital malformation of choroid |
| Q14.8 | Other congenital malformations of posterior segment of eye |
| Q14.8 | Coloboma of fundus |
| Q14.9 | Congenital malformation of posterior segment of eye, unspecified |
| Q15 | Other congenital malformations of eye |
| Q15.0 | Congenital glaucoma |
| Q15.0 | Buphthalmos |
| Q15.0 | Glaucoma of newborn |
| Q15.0 | Hydrophthalmos |
| Q15.0 | Keratoglobus, congenital with glaucoma |
| Q15.0 | Megalocornea with glaucoma |
| Q15.0 | Macrophthalmos in congenital glaucoma |
| Q15.0 | Macrocornea wit glaucoma |
| Q15.8 | Other specified congenital malformations of eye |
| Q15.9 | Other congenital malformation of eye, unspecified |
| Q15.9 | Congenital anomaly of eye NOS |
| Q15.9 | Congenital deformity of eye NOS |
| Q16 | Congenital malformations of ear causing impairment of hearing |
| Q16.0 | Congenital absence of(ear) auricle |
| Q16.1 | Congenital absence, atresia and stricture of auditory canal(external) |
| Q16.1 | Atresia or stricture of osseous meatus |
| Q16.2 | Absence of eustachian tube |
| Q16.3 | Congenital malformation of ear ossicles |
| Q16.3 | Fusion of ear ossicles |
| Q16.4 | Other congenital malformations of middle ear |
| Q16.4 | Congenital malformation of middle ear NOS |
| Q16.5 | Congenital malformation of inner ear |
| Q16.5 | Anomaly of membranous labyrinth |
| Q16.5 | Anomaly of Corti organ |
| **ICD-10 Code,  with subsections** | **Term** |
| Q16.9 | Congenital malformation of ear causing impairment of hearing, unspecified |
| Q16.9 | Congenital absence of ear NOS |
| Q20 | Congenital malformations of cardiac chambers and connections |
| Q20.0 | Common arterial trunk |
| Q20.0 | Persistent truncus arteriosus |
| Q20.1 | Double outlet right ventricle |
| Q20.1 | Taussig-Bing syndrome |
| Q20.2 | Double outlet left ventricle |
| Q20.3 | Discordant ventriculoarterial connection |
| Q20.3 | Dextrotransposition of aorta |
| Q20.3 | Transposition of great vessels(complete) |
| Q20.4 | Double inlet ventricle |
| Q20.4 | Common ventricle |
| Q20.4 | Cor triloculare biatriatum |
| Q20.4 | Single ventricle |
| Q20.5 | Discordant atrioventricular connection |
| Q20.5 | Corrected transposition |
| Q20.5 | Laevotransposition |
| Q20.5 | Ventricular inversion |
| Q20.6 | Isomerism of atrial appendages |
| Q20.6 | Isomerism of atrial appendages with asplenia or polysplenia |
| Q20.8 | Other congenital malformations of cardiac chambers and connections |
| Q20.9 | Congenital malformation of cardiac chambers and connections, unspecified |
| Q21.9 | Ventricular septal defect, unspecified |
| Q21.2 | Atrioventricular septal defect |
| Q21.2 | Common atrioventricular canal |
| Q21.2 | Endocardial cushion defect |
| Q21.2 | Ostium primum atrial septal defect (typeⅠ) |
| Q21.3 | Tetralogy of Fallot |
| Q21.3 | Ventricular septal defect with pulmonary stenosis or atresia, dextroposition of aorta and hypertrophy of rigth ventricle |
| Q21.4 | Aortopulmonary septal defect |
| Q21.4 | Aortic septal defect |
| Q21.4 | Aortopulmonary window |
| Q21.8 | Other congenital malformations of cardiac septa |
| Q21.8 | Eisenmenger's defect |
| Q21.8 | Pentalogy of Fallot |
| Q21.9 | Congenital malformation of cardiac septum, unspecified |
| Q21.9 | Septal (heart) defect NOS |
| Q22 | Congenital malformations of pulmonary and tricuspid valves |
| Q22.0 | Pulmonary valve atresia |
| Q22.1 | Congenital pulmonary valve stenosis |
| Q22.2 | Congenital pulmonary valve insufficiency |
| Q22.2 | Congenital pulmonary valve regurgitation |
| Q22.3 | Other congenital malformations of pulmonary valve |
| Q22.3 | Congenital malformation of pulmonary valve NOS |
| **ICD-10 Code,  with subsections** | **Term** |
| Q22.4 | Congenital tricuspid stenosis |
| Q22.4 | Tricuspid atresia |
| Q22.5 | Ebstein's anomaly |
| Q22.6 | Hypoplastic right heart syndrome |
| Q22.8 | Other congenital malformations of tricuspid valve |
| Q22.9 | Congenital malformation of tricuspid valve, unspecified |
| Q23 | Congenital malformations of aortic and mitral valves |
| Q23.0 | Congenital stenosis of aortic valve |
| Q23.0 | Congenital aortic atresia |
| Q23.0 | Congenital aortic stenosis |
| Q23.1 | Congenital insufficiency of aortic valve |
| Q23.1 | Bicuspid aortic valve |
| Q23.1 | Congenital aortic insufficiency |
| Q23.2 | Congenital mitral stenosis |
| Q23.2 | Congenital mitral atresia |
| Q23.3 | Congenital mitral insufficiency |
| Q23.4 | Hypoplastic left heart syndrome |
| Q23.4 | Atresia, or marked hypoplasia of aortic orifice or valve, with hypoplasia of ascending aorta and defective development of left ventricle (with mitral valve stenosis or atresia) |
| Q23.8 | Other congenital malformations of aortic and mitral valves |
| Q23.9 | Congenital malformation of aortic and mitral valves, unspecified |
| Q24 | Other congenital malformations of heart |
| Q24.2 | Cor triatriatum |
| Q24.3 | Pulmonary infundibular stenosis |
| Q24.4 | Congenital subaortic stenosis |
| Q24.5 | Malformation of coronary vessels |
| Q24.5 | Congenital coronary(artery) aneurysm |
| Q24.6 | Congenital heart block |
| Q24.8 | Other specified congenital malformations of heart |
| Q24.8 | Congenital diverticulum of left ventricle |
| Q24.8 | Congenital malformation of myocardium |
| Q24.8 | Congenital malformation of pericardium |
| Q24.8 | Malposition of heart |
| Q24.8 | Uhl's disease |
| Q25 | Congenital malformations of great arteries |
| Q25.1 | Coarctation of aorta |
| Q25.1 | Coarctation of aorta (preductal, postductal) |
| Q25.2 | Atresia of aorta |
| Q25.3 | Stenosis of aorta |
| Q25.3 | Supravalvular aortic stenosis |
| Q25.4 | Other congenital malformations of aorta |
| Q25.4 | Absence of aorta |
| Q25.4 | Aplasia of aorta |
| Q25.4 | Congenital aneurysm of aorta |
| Q25.4 | Congenital dilatation of aorta |
| Q25.4 | Aneurysm of sinus of Valsalva(ruptured) |
| **ICD-10 Code,  with subsections** | **Term** |
| Q25.4 | Double aortic arch[vascular ring of aorta] |
| Q25.4 | Hypoplasia of aorta |
| Q25.4 | Persistent convolutions of aortic arch |
| Q25.4 | Persistent right aortic arch |
| Q25.5 | Atresia of pulmonary artery |
| Q25.6 | Stenosis of pulmonary artery |
| Q25.6 | Supravalvular pulmonary stenosis |
| Q25.7 | Other congenital malformations of pulmonary artery |
| Q25.7 | Aberrant pulmonary artery |
| Q25.7 | Agenesis of pulmonary artery |
| Q25.7 | Aneurysm of pulmonary artery |
| Q25.7 | Anomaly of pulmonary artery |
| Q25.7 | Hypoplasia of pulmonary artery |
| Q25.7 | Pulmonary arteriovenous aneurysm |
| Q25.8 | Other congenital malformations of great arteries |
| Q25.9 | Congenital malformation of great arteries, unspecified |
| Q26 | Congenital malformations of great veins |
| Q26.0 | Congenital stenosis of vena cava |
| Q26.0 | Congenital stenosis vena cava (inferior, superior) |
| Q26.1 | Persistent left superior vena cava |
| Q26.2 | Total anomalous pulmonary venous connection |
| Q26.3 | Partial anomalous pulmonary venous connection |
| Q26.4 | Anomalous pulmonary venous connection, unspecified |
| Q26.5 | Anomalous portal venous connection |
| Q26.6 | Portal vein-hepatic artery fistula |
| Q26.8 | Other congenital malformations of great veins |
| Q26.8 | Absence of vena cava (inferior, superior) |
| Q26.8 | Azygos continuation of inferior vena cava |
| Q26.8 | Persistent left posterior cardinal vein |
| Q26.8 | Scimitar syndrome |
| Q26.9 | Congenital malformation of great vein, unspecified |
| Q26.9 | Anomaly of vena cava (inferior, superior) NOS |
| Q27 | Other congenital malformations of peripheral vascular system |
| Q27.0 | Congenital absence and hypoplasia of umbilical artery |
| Q27.1 | Congenital renal artery stenosis |
| Q27.2 | Other congenital malformations of renal artery |
| Q27.2 | Congenital malformation of renal artery NOS |
| Q27.2 | Multiple renal arteries |
| Q27.3 | Peripheral arteriovenous malformation |
| Q27.3 | Arteriovenous aneurysm |
| Q27.4 | Congenital phlebectasia |
| Q27.8 | Other specified congenital malformations of peripheral vascular system |
| Q27.8 | Aberrant subclavian artery |
| Q27.8 | Absence of artery or vein NEC |
| Q27.8 | Atresia of artery or vein NEC |
| Q27.8 | Congenital aneurysm, peripheral |
| **ICD-10 Code,  with subsections** | **Term** |
| Q27.8 | Congenital stricture, artery |
| Q27.8 | Congenital varix |
| Q27.9 | Congenital malformation of peripheral vascular system, unspecified |
| Q27.9 | Anomaly of artery or vein NOS |
| Q28 | Other congenital malformations of circulatory system |
| Q28.0 | Arteriovenous malformation of precerebral vessels |
| Q28.0 | Congenital arteriovenous precerebral aneurysm(nonruptured) |
| Q28.1 | Other malformations of precerebral vessels |
| Q28.1 | Congenital malformation of precerebral vessels NOS |
| Q28.1 | Congenital precerebral aneurysm (nonruptured) |
| Q28.2 | Arteriovenous malformation of cerebral vessels |
| Q28.2 | Arteriovenous malformation of brain NOS |
| Q28.2 | Congenital arteriovenous cerebral aneurysm (nonruptured) |
| Q28.3 | Other malformation of cerebral vessels |
| Q28.3 | Congenital cerebral aneurysm (nonruptured) |
| Q28.3 | Congenital malformation of cerebral vessels, NOS |
| Q28.8 | Other specified congenital malformations of circulatory system |
| Q28.8 | Congenital aneurysm specified site NEC |
| Q28.9 | Congenital malformations of circulatory system, unspecified |
| Q30 | Congenital malformations of nose |
| Q30.0 | Choanal atresia |
| Q30.0 | Atresia of nares (anterior, posterior) |
| Q30.0 | Congenital stenosis of nares (anterior, posterior) |
| Q30.1 | Agenesis and underdevelopment of nose |
| Q30.1 | Congenital absence of nose |
| Q30.2 | Fissured, notched and cleft nose |
| Q30.3 | Congenital perforated nasal septum |
| Q30.8 | Other congenital malformations of nose |
| Q30.8 | Accessory nose |
| Q30.8 | Congenital anomaly of nasal sinus wall |
| Q30.9 | Congenital malformations of nose, unspecified |
| Q31 | Congenital malformations of larynx |
| Q31.0 | Web of larynx |
| Q31.0 | NOS web of larynx |
| Q31.0 | Glottic web of larynx |
| Q31.0 | Subglottic web of larynx |
| Q31.1 | Congenital subglottic stenosis |
| Q31.2 | Laryngeal hypoplasia |
| Q31.3 | Laryngocele |
| Q31.5 | Congenital laryngomalacia |
| Q31.8 | Other congenital malformations of larynx |
| Q31.8 | Absence of cricoid cartilage, epiglottis, glottis, larynx or thyroid cartilage |
| Q31.8 | Agenesis of cricoid cartilage, epiglottis, glottis, larynx or thyroid cartilage |
| Q31.8 | Atresia of cricoid cartilage, epiglottis, glottis, larynx or thyroid cartilage |
| Q31.8 | Cleft thyroid cartilage |
| Q31.8 | Congenital stenosis of larynx NEC |
| **ICD-10 Code,  with subsections** | **Term** |
| Q31.8 | Fissure of epiglottis |
| Q31.8 | Posterior cleft of cricoid cartilage |
| Q31.9 | Congenital malformation of larynx, unspecified |
| Q32 | Congenital malformations of trachea and bronchus |
| Q32.0 | Congenital tracheomalacia |
| Q32.1 | Other congenital malformations of trachea |
| Q32.1 | Anomaly of tracheal cartilage |
| Q32.1 | Atresia of trachea |
| Q32.1 | Congenital dilatation (of) trachea |
| Q32.1 | Congenital malformation (of) trachea |
| Q32.1 | Congenital stenosis (of) trachea |
| Q32.1 | Congenital tracheocele |
| Q32.2 | Congenital bronchomalacia |
| Q32.3 | Congenital stenosis of bronchus |
| Q32.4 | Other congenital malformations of bronchus |
| Q32.4 | Absence of bronchus |
| Q32.4 | Agenesis of bronchus |
| Q32.4 | Atresia of bronchus |
| Q32.4 | Congenital malformation NOS of bronchus |
| Q32.4 | Diverticulum of bronchus |
| Q33 | Congenital malformations of lung |
| Q33.0 | Congenital cystic lung |
| Q33.0 | Congenital honeycomb lung |
| Q33.0 | Congenital cystic lung disease |
| Q33.0 | Congenital polycystic lung disease |
| Q33.1 | Accessory lobe of lung |
| Q33.2 | Sequestration of lung |
| Q33.3 | Agenesis of lung |
| Q33.3 | Absecne of lung (lobe) |
| Q33.4 | Congenital bronchiectasis |
| Q33.5 | Ectopic tissue in lung |
| Q33.6 | Hypoplasia and dysplasia of lung |
| Q33.8 | Other congenital malformations of lung |
| Q33.9 | Congenital malformation of lung, unspecified |
| Q34 | Other congenital malformations of respiratory system |
| Q34.0 | Anomaly of pleura |
| Q34.1 | Congenital cyst of mediastinum |
| Q34.8 | Other specified congenital malformations of respiratory system |
| Q34.8 | Atresia of nasopharynx |
| Q34.9 | Congenital malformation of respiratory system, unspecified |
| Q34.9 | Congenital absence of respiratory organ |
| Q34.9 | Congenital anomaly of respiratory organ NOS |
| Q35 | Cleft palate |
| Q35 | Fissure of palate |
| Q35 | Palatoschisis |
| Q35.1 | Cleft hard palate |
| **ICD-10 Code,  with subsections** | **Term** |
| Q35.3 | Cleft soft palate |
| Q35.5 | Cleft hard palate with cleft soft palate |
| Q35.7 | Cleft uvula |
| Q35.9 | Cleft palate, unspecified |
| Q35.9 | Cleft palate, NOS |
| Q37 | Cleft palate with cleft lip |
| Q37.0 | Cleft hard palate with bilateral cleft lip |
| Q37.1 | Cleft hard palate with unilateral cleft lip |
| Q37.1 | Cleft hard palate with cleft lip NOS |
| Q37.2 | Cleft soft palate with bilateral cleft lip |
| Q37.3 | Cleft soft palate with unilateral cleft lip |
| Q37.3 | Cleft soft palate with cleft lip NOS |
| Q37.4 | Cleft hard and soft palate with bilateral cleft lip |
| Q37.5 | Cleft hard and soft palate with unilateral cleft lip |
| Q37.5 | Cleft hard and soft palate with cleft lip NOS |
| Q37.8 | Unspecified cleft palate with bilateral cleft lip |
| Q37.9 | Unspecified cleft palate with unilateral cleft lip |
| Q37.9 | Cleft palate with cleft lip NOS |
| Q38 | Other congenital malformations of tongue, mouth and pharynx |
| Q38.0 | Cogenital malformations of lips, NEC |
| Q38.0 | Abnormal labia frenum |
| Q38.8 | Other congenital malformations of lips, NEC |
| Q38.8 | Cogenital fistula of lips NEC |
| Q38.8 | Cogenital malformations of lip NOS |
| Q38.8 | Van der Woude's syndrome |
| Q38.2 | Macroglossia |
| Q38.3 | Other congenital malformations of tongue |
| Q38.3 | Aglossia |
| Q38.3 | Bifid tongue |
| Q38.3 | Congenital adhesion of tongue |
| Q38.3 | Congenital fissure of tongue |
| Q38.3 | Congenital malformation of tongue NOS |
| Q38.3 | Hypoplasia of tongue |
| Q38.3 | Hypoglossia |
| Q38.3 | Microglossia |
| Q38.4 | Congenital malformations of salivary glands and ducts |
| Q38.4 | Absence (of) salivary gland or duct |
| Q38.4 | Accessory (of) salivary gland or duct |
| Q38.4 | Atresia (of) salivary gland or duct |
| Q38.4 | Congenital fistula of salivary glands |
| Q38.5 | Congenital malformations of palate, NEC |
| Q38.5 | Absence of uvula |
| Q38.5 | Congenital malformations of palate NOS |
| Q38.5 | High arched palate |
| Q38.6 | Other congenital malformation of mouth |
| Q38.6 | Congenital malformation of mouth NOS |
| **ICD-10 Code,  with subsections** | **Term** |
| Q38.7 | Pharyngeal pouch |
| Q38.7 | Diverticulum of pharynx |
| Q38.8 | Other congenital malformations of pharynx |
| Q38.80 | Velopharyngeal insufficiency |
| Q38.88 | Other congenital malformations of pharynx |
| Q39 | Congenital malformations of oesophagus |
| Q39.0 | Atresia of oesophagus without fistula |
| Q39.0 | Atresia of oesophagus NOS |
| Q39.1 | Atresia of oesophagus with tracheo-esophageal fistula |
| Q39.1 | Atresia of oesophagus with broncho-esophageal fistula |
| Q39.2 | Congenital tracheo-esophageal fistula without atresia |
| Q39.2 | Congenital tracheo-esophageal fistula NOS |
| Q39.3 | Congenital stenosis and stricture of oesophagus |
| Q39.4 | Esophageal web |
| Q39.5 | Congenital dilatation of oesophagus |
| Q39.6 | Diverticulum of oesophagus |
| Q39.6 | Esophageal pouch |
| Q39.8 | Other congenital malformations of oesophagus |
| Q39.8 | Absent (of) oesophagus |
| Q39.8 | Congenital displacement (of) oesophagus |
| Q39.8 | Duplication (of) oesophagus |
| Q39.9 | Congenital malformation of oesophagus, unspecified |
| Q40.1 | Congenital hiatus hernia |
| Q40.1 | Displacement of cardia through esophageal hiatus |
| Q40.2 | Other specified congenital malformations of stomach |
| Q40.2 | Congenital displacement of stomach |
| Q40.2 | Congenital diverticulum of stomach |
| Q40.2 | Congenital hourglass stomach |
| Q40.2 | Duplication of stomach |
| Q40.2 | Megalogastria |
| Q40.2 | Microgastria |
| Q40.3 | Congenital malformation of stomach, unspecified |
| Q40.8 | Other specified congenital malformations of upper alimentary tract |
| Q40.9 | Congenital malformation of upper alimentary tract, unspecified |
| Q40.9 | Congenital anomaly of upper alimentary tract NOS |
| Q40.9 | Congenital deformity of upper alimentary tract NOS |
| Q41 | Congenital absence, atresia and stenosis of small intestine |
| Q41 | Congenital obstruction, occlusion and stricture of small intestine or intestine NOS |
| Q41.0 | Congenital absence, atresia and stenosis of duodenum |
| Q41.1 | Congenital absence, atresia and stenosis of jejunum |
| Q41.1 | Apple peel syndrome |
| Q41.1 | Imperforate jejunum |
| Q41.2 | Congenital absence, atresia and stenosis of ileum |
| Q41.8 | Congenital absence, atresia and stenosis of other specified parts of small intestine |
| Q41.9 | Congenital absence, atresia and stenosis of small intestine, part unspecified |
| **ICD-10 Code,  with subsections** | **Term** |
| Q41.9 | Congenital absence, atresia and stenosis of intestine NOS |
| Q42 | Congenital absence, atresia and stenosis of large intestine |
| Q42 | Congenital obstruction, occlusion and stricture of large intestine |
| Q42.0 | Congenital absence, atresia and stenosis of rectum with fistula |
| Q42.1 | Congenital absence, atresia and stenosis of rectum without fistula |
| Q42.1 | Imperforate rectum |
| Q42.2 | Congenital absence, atresia and stenosis of anus with fistula |
| Q42.3 | Congenital absence, atresia and stenosis of anus without fistula |
| Q42.3 | Imperforate anus |
| Q42.8 | Congenital absence, atresia and stenosis of other parts of large intestine |
| Q42.9 | Congenital absence, atresia and stenosis of large intestine, part unspecified |
| Q43 | Other congenital malformations of intestine |
| Q43.0 | Meckel's diverticulum |
| Q43.0 | Persistent omphalomesenteric duct |
| Q43.0 | Persistent vitelline duct |
| Q43.1 | Hirschsprung's disease |
| Q43.1 | Aganglionosis |
| Q43.1 | Congenital (aganglionic) megacolon |
| Q43.2 | Other congenital functional disorders of colon |
| Q43.2 | Congenital dilatation of colon |
| Q43.3 | Congenital malformations of intestinal fixation |
| Q43.3 | Congenital anomalous omental adhesions[bands] |
| Q43.3 | Congenital peritoneal adhesions[bands] |
| Q43.3 | Jackson's membrane |
| Q43.3 | Malrotation of colon |
| Q43.3 | Failure of rotation of cecum and colon |
| Q43.3 | Incomplet rotation of cecum and colon |
| Q43.3 | Insufficient rotation of cecum and colon |
| Q43.3 | Universal mesentary |
| Q43.4 | Duplication of intestine |
| Q43.5 | Ectopic anus |
| Q43.6 | Congenital fistula of rectum and anus |
| Q43.7 | Persistent cloaca |
| Q43.7 | Cloaca NOS |
| Q43.8 | Other specified congenital malformations of intestine |
| Q43.8 | Congenital blind loop syndrome |
| Q43.8 | Congenital diverticulitis, colon |
| Q43.8 | Congenital diverticulum, intestine |
| Q43.8 | Dolichocolon |
| Q43.8 | Megaloappendix |
| Q43.8 | Megaloduodenum |
| Q43.8 | Transposition of Microcolon |
| Q43.8 | Transposition of appendix |
| Q43.8 | Transposition of colon |
| Q43.8 | Transposition of intestine |
| Q43.9 | Congenital malformation of intestine, unspecified |
| **ICD-10 Code,  with subsections** | **Term** |
| Q44 | Congenital malformations of gallbladder, bile ducts and liver |
| Q44.0 | Agenesis, aplasia and hypoplasia of gallbladder |
| Q44.0 | Congenital absence of gallbladder |
| Q44.1 | Other congenital malformations of gallbladder |
| Q44.1 | Congenital malformations of gallbladder NOS |
| Q44.1 | Intrahepatic gallbladder |
| Q44.2 | Atresia of bile ducts |
| Q44.3 | Congenital stenosis and stricture of bile ducts |
| Q44.4 | Choledochal cyst |
| Q44.5 | Other congenital malformations of biliary ducts |
| Q44.5 | Accessory hepatic duct |
| Q44.5 | Congenital malformations of bile duct NOS |
| Q44.5 | Duplication of bile duct |
| Q44.5 | Duplication of cystic duct |
| Q44.6 | Cystic disease of liver |
| Q44.6 | Fibrocystic disease of liver |
| Q44.7 | Other congenital malformations of liver |
| Q44.7 | Accessory liver |
| Q44.7 | Alagille's syndrome |
| Q44.7 | Congenital absence of liver |
| Q44.7 | Congenital hepatomegaly |
| Q44.7 | Congenital malformation of liver NOS |
| Q45 | Other congenital malformations of digestive system |
| Q45.0 | Agenesis, aplasia and hypoplasia of pancreas |
| Q45.0 | Congenital absence of pancreas |
| Q45.1 | Annular pancreas |
| Q45.2 | Congenital pancreatic cyst |
| Q45.3 | Other congenital malformations of pancreas and pancreatic duct |
| Q45.3 | Accessory pancreas |
| Q45.3 | congenital malformations of pancreas or pancreatic duct nos |
| Q45.8 | Other specified congenital malformations of digestive system |
| Q45.8 | Absence of alimentary tract(complete, partial) NOS |
| Q45.8 | Duplication of digestive organs NOS |
| Q45.8 | Malposition of digestive organs, congenital NOS |
| Q45.9 | Congenital malformation of digestive system, unspecified |
| Q45.9 | Congenital anomaly of digestive system NOS |
| Q45.9 | Congenital deformity of digestive system NOS |
| Q60 | Renal agenesis and other reduction defects of kidney |
| Q60 | Congenital atrophy of kidney |
| Q60 | Infantile atrophy of kidney |
| Q60 | Congenitla absence of kidney |
| Q60.0 | Renal agenesis, unilateral |
| Q60.1 | Renal agenesis, bilateral |
| Q60.2 | Renal agenesis, unspecified |
| Q60.3 | Renal hypoplasia, unilateral |
| Q60.4 | Renal hypoplasia, bilateral |
| **ICD-10 Code,  with subsections** | **Term** |
| Q60.5 | Renal hypoplasia, unspecified |
| Q60.6 | Potter's syndrome |
| Q61 | Cystic kidney disease |
| Q61.1 | Polycystic kidney, autosomal recessive |
| Q61.1 | Polycystic kidney, infantile type |
| Q61.2 | Polycystic kidney, autosomal dominant |
| Q61.2 | Polycystic kidney, adult type |
| Q61.3 | Polycystic kidney, unspecified |
| Q61.4 | Renal dysplasia |
| Q61.4 | Multicystic dyplastic kidney |
| Q61.4 | Multicystic kidney (developmental) |
| Q61.4 | Multicystic kidney disease |
| Q61.4 | Multicystic renal dysplasia |
| Q61.5 | Medullary cystic kidney |
| Q61.5 | Sponge kidney NOS |
| Q61.8 | Other cystic kidney diseases |
| Q61.8 | Fibrocystic kidney |
| Q61.8 | Fibrocystic renal degeneration or disease |
| Q61.9 | Cystic kidney disease, unspecified |
| Q61.9 | Meckel-Gruber syndrome |
| Q76 | Congenital malformations of spine and bony thorax |
| Q76.0 | Spina bifida occulta |
| Q76.1 | Klippel-Feil syndrome |
| Q76.1 | Cervical fusion syndrome |
| Q76.2 | Congenital spondylolisthesis |
| Q76.2 | Congenital spondylolysis |
| Q76.3 | Congenital scoliosis due to congenital bony malformation |
| Q76.3 | Hemivertebra fusion or failure of segmentation with scoliosis |
| Q76.4 | Other congenital malformations of spine, not associated with scoliosis |
| Q76.4 | Congenital absence of vertebra unspecified or not associated with scoliosis |
| Q76.4 | Congenital fusion of spine unspecified or not associated with scoliosis |
| Q76.4 | Congenital kyphosis unspecified or not associated with scoliosis |
| Q76.4 | Congenital lordosis unspecified or not associated with scoliosis |
| Q76.4 | Congenital malformation of lumbosacral (joint) (region) unspecified or not associated with scoliosis |
| Q76.4 | Hemivertebra unspecified or not associated with scoliosis |
| Q76.4 | Malformation of spine unspecified or not associated with scoliosis |
| Q76.4 | Platyspondylisis unspecified or not associated with scoliosis |
| Q76.4 | Supernumerary vertebra unspecified or not associated with scoliosis |
| Q76.5 | Congenital malformations of cervical rib |
| Q76.5 | Supernumerary rib in cervical region |
| Q76.6 | Other congenital malformations of ribs |
| Q76.6 | Accessory rib |
| Q76.6 | Congenital absence of rib |
| Q76.6 | Congenital fusion of ribs |
| Q76.6 | Congenital malformation of ribs NOS |
| **ICD-10 Code,  with subsections** | **Term** |
| Q76.7 | Congenital malformation of sternum |
| Q76.7 | Congenital absence of sternum |
| Q76.7 | Sternum bifidum |
| Q76.8 | Other congenital malformations of bony thorax |
| Q76.9 | Congenital malformation of bony thorax, unspecified |
| Q77 | Osteochondrodysplasia with defects of growth of tubular bones and spine |
| Q77.0 | Achondrogenesis |
| Q77.0 | Hypochondrogenesis |
| Q77.1 | Thanatophoric short stature |
| Q77.2 | Short rib syndrome |
| Q77.2 | Asphyxiating thoracic dysplasia[Jeune] |
| Q77.3 | Chondrodysplasia punctata |
| Q77.4 | Achondroplasia |
| Q77.4 | Hypochondroplasia |
| Q77.4 | congenita osteosclerosis |
| Q77.5 | Diastrophic dysplasia |
| Q77.6 | Chondroectodermal dyplasia |
| Q77.6 | Ellis-van Creveld syndrome |
| Q77.7 | Spondyloepiphyseal dysplasia |
| Q77.8 | Other osteochondrodysplasia with defects of growth of tubular bones and spine |
| Q77.9 | Osteochondrodysplasia with defects of growth of tubular bones and spine, unspecified |
| Q78 | Other osteochondrodysplasias |
| Q78.0 | Osteogenesis imperfecta |
| Q78.0 | Fragilitas ossium |
| Q78.0 | Osteopsathyrosis |
| Q78.1 | Polyostotic fibrous dysplasia |
| Q78.1 | Albright(-McCune)(-Sternberg) syndrome |
| Q78.2 | Osteopetrosis |
| Q78.2 | Albers-Schonberg syndrome |
| Q78.3 | Progressive diaphyseal dysplasia |
| Q78.3 | Camurati-Engelmann syndrome |
| Q78.4 | Enchondromatosis |
| Q78.4 | Maffucci's syndrome |
| Q78.4 | Ollier's disease |
| Q78.5 | Metaphyseal dysplasia |
| Q78.5 | Pyle's syndrome |
| Q78.6 | Multiple congenital exostoses |
| Q78.6 | Diaphyseal aclasis |
| Q78.8 | Other specified osteochondrodysplasias |
| Q78.8 | Osteopoikilosis |
| Q78.9 | Osteochondrodysplasia, unspecified |
| Q78.9 | Chondrodystrophy NOS |
| Q78.9 | Osteodystrophy NOS |
| Q79 | Congenital malformations of musculoskeletal system, NEC |
| Q79.0 | Congenital diaphragmatic hernia |
| **ICD-10 Code,  with subsections** | **Term** |
| Q79.1 | Other congenital malformations of diaphragm |
| Q79.1 | Absence of diaphragm |
| Q79.1 | Congenital malformations of diaphragm NOS |
| Q79.1 | Eventration of diaphragm |
| Q79.2 | Exomphalos |
| Q79.2 | Omphalocele |
| Q79.3 | Gastroschisis |
| Q79.4 | Prune belly syndrome |
| Q79.5 | Other congenital malformations of abdominal wall |
| Q79.6 | Ehlers-Danlos syndrome |
| Q79.8 | Other congenital malformations of musculoskeletal system |
| Q79.8 | Absence of muscle |
| Q79.8 | Absence of tendon |
| Q79.8 | Accessory muscle |
| Q79.8 | Amyotrophia congenita |
| Q79.8 | Congenital constriting bands |
| Q79.8 | Congenital shortening of tendon |
| Q79.8 | Poland's syndrome |
| Q79.9 | Congenital malformation of musculoskeletal system, unspecified |
| Q79.9 | Congenital anomaly of musculoskeletal system NOS |
| Q79.9 | Congenital deformity of musculoskeletal system NOS |
| Q86.0 | Fetal alcohol syndrome (dysmorphic) |
| Q86.1 | Fetal hydantoin syndrome |
| Q86.1 | Meadow's syndrome |
| Q86.2 | Dysmorphism due to warfarin |
| Q86.8 | Other congenital malformation syndromes due to known exogenous causes |
| Q87 | Other specified congenital malformation syndromes affecting multiple systems |
| Q87.0 | Congenital malformation syndromes predominantly affecting facial appearance |
| Q87.0 | Acrocephalopolysyndactyly |
| Q87.0 | Acrocephalosyndactyly (Apert) |
| Q87.0 | Cryptophthalmos syndrome |
| Q87.0 | Cyclopia syndrome |
| Q87.0 | Goldenhar syndrome |
| Q87.0 | Moebius syndrome |
| Q87.0 | Oro-facial-digital syndrome |
| Q87.0 | Robin syndrome |
| Q87.0 | Whistling face |
| Q87.1 | Congenital malformation syndromes predominantly associated with short stature |
| Q87.1 | Aarskog syndrome |
| Q87.1 | cockayne syndrome |
| Q87.1 | De lange syndrome |
| Q87.1 | Dubowitz syndrome |
| Q87.1 | Noonan syndrome |
| Q87.1 | Prader-Willi syndrome |
| Q87.1 | Robinow-Silverman-Smith syndrome |
| Q87.1 | Russel-Silver syndrome |
| **ICD-10 Code,  with subsections** | **Term** |
| Q87.1 | Seckel syndrome |
| Q87.1 | Smith-Lemli-Opitz syndrome |
| Q87.2 | Congenital malformation syndromes predominantly involving limbs |
| Q87.2 | Holt-Oram syndrome |
| Q87.2 | Klippel-Trenaunay-weber syndrome |
| Q87.2 | Nail patella syndrome |
| Q87.2 | Rubinstein-Taybi syndrome |
| Q87.2 | Sirenomelia syndrome |
| Q87.2 | Thrombocytopenia with absent radius[TAR] syndrome |
| Q87.2 | VATER syndrome |
| Q87.3 | Congenital malformation syndromes involving early overgrowth |
| Q87.3 | Beckwith-Wiedmann syndrome |
| Q87.3 | Sotos syndrome |
| Q87.3 | Weaver syndrome |
| Q87.4 | Marfan's syndrome |
| Q87.5 | Other congenital malformation syndromes with other skeletal changes |
| Q87.8 | Other specified congenital malformation syndromes, NEC |
| Q87.8 | Alport syndrome |
| Q87.8 | Laurence-Moon(-Bardet)-Biedl syndrome |
| Q87.8 | Zellweger syndrome |
| Q87.8 | CHARGE syndrome |
| Q89 | Other congenital malformations, NEC |
| Q89.0 | Congenital malformations of spleen |
| Q89.0 | Asplenia (congenital) |
| Q89.8 | Other specified congenital malformations of spleen |
| Q89.8 | Congenital splenomegaly |
| Q89.4 | Conjoined twins |
| Q89.4 | Craniopagus |
| Q89.4 | Dicephaly |
| Q89.4 | Double monster |
| Q89.4 | Pygopagus |
| Q89.4 | Thoracopagus |
| Q89.7 | Multiple congenital malformations, NEC |
| Q89.7 | Monster NOS |
| Q89.7 | Multiple congenital anomalies NOS |
| Q89.7 | Multiple congenital deformities NOS |
| Q89.8 | Other specified congenital malformations |
| Q89.9 | Congenital malformation, unspecified |
| Q89.9 | Congenital anomaly NOS |
| Q89.9 | Congenital deformity NOS |
| Q90 | Down's syndrome |
| Q90.0 | Trisomy 21, meiotic nondisjunction |
| Q90.1 | Trisomy 21, mosaicism (mitotic nondisjunction) |
| Q90.2 | Trisomy 21, translocation |
| Q90.9 | Down's syndrome, unspecified |
| Q90.9 | Trisomy 21 NOS |
| **ICD-10 Code,  with subsections** | **Term** |
| Q91 | Edwards's syndrome and patau's syndrome |
| Q91.0 | Trisomy 18, meiotic nondisjunction |
| Q91.1 | Trisomy 18, mosaicism, mitotic nondisjunction |
| Q91.2 | Trisomy 18, translocation |
| Q91.3 | Edwards's syndrome, unspecified |
| Q91.4 | Trisomy 13, meiotic nondisjunction |
| Q91.5 | Trisomy 13, mosaicism (mitotic nondisjunction) |
| Q91.6 | Trisomy 13, translocation |
| Q91.7 | Patau's syndrome, unspecified |
| Q92 | Other trisomies and partial trisomies of the autosomes, NEC |
| Q92 | Unbalanced translocations and insertions |
| Q92.0 | Whole chromosome trisomy, meiotic nondisjunction |
| Q92.1 | Whole chromosome trisomy, mosaicism (mitotic nondisjunction) |
| Q92.2 | Major partial trisomy |
| Q92.2 | Whole arm or more duplicated |
| Q92.3 | Minor partial trisomy |
| Q92.3 | Less than whole arm duplicated |
| Q92.4 | Duplications seen only at prometaphase |
| Q92.5 | Duplications with other complex rearrangements |
| Q92.6 | Extra marker chromosomes |
| Q92.7 | Triploidy and polyploidy |
| Q92.8 | Other specified trisomies and partial trisomies of ther autosomes |
| Q92.9 | Trisomy and partial trisomy of ther autosomes, unspecified |
| Q93 | Monosomies and deletions from the autosomes, NEC |
| Q93.0 | Whole chromosome monosomy, meiotic nondisjunction |
| Q93.1 | Whole chromosome monosomy, mosaicism (mitotic nondisjunction) |
| Q93.2 | Chromosome replaced with ring or dicentric |
| Q93.3 | Deletion of short arm of chromosome4 |
| Q93.3 | Wolff-Hirschorn syndrome |
| Q93.4 | Deletion of short arm of chromosome 5 |
| Q93.4 | Cri-du-chat syndrome |
| Q93.5 | Other deletions of part of a chromosome |
| Q93.5 | Angelman syndrome |
| Q93.5 | CATCH22 syndrome |
| Q93.6 | Deletions seen only at prometaphase |
| Q93.7 | Deletions with other complex rearrangements |
| Q93.8 | Other deletions from the autosomes |
| Q93.9 | Deletions from autosomes, unspecified |
| Q96 | Turner's syndrome |
| Q96.0 | Karyotype 45, X |
| Q96.1 | Karyotype 46, X iso(Xq) |
| Q96.2 | Karyotype 46,X with abnormal sex chromosome, except iso(Xq) |
| Q96.3 | Mosaicism, 45, X/46, XX or XY |
| Q96.4 | Mosaicism, 45, X/other cell line(s) with abnormal sex chromosome |
| Q96.8 | Other variants of Turner's syndrome |
| Q96.9 | Turner's syndrome, unspecified |
| **ICD-10 Code,  with subsections** | **Term** |
| Q97 | Other sex chromosome abnormalities, female phenotype, NEC |
| Q97.0 | Karyotype 47, XXX |
| Q97.1 | Female with more than three X chromosomes |
| Q97.2 | Mosaicism, lines with various numbers of X chromosomes |
| Q97.3 | Female with 46, XY karyotype |
| Q97.8 | Other specified sex chromosome abnormalities, female phenotype |
| Q97.9 | Sex chromosome abnormality, female phenotype, unspecified |
| Q98 | Other sex chromosome abnormalities, male phenotype, NEC |
| Q98.0 | Klinefelter's syndrome karyotype 47, XXY |
| Q98.1 | Klinefelter's syndrome, male with more than two X chromosomes |
| Q98.2 | Klinefelter's syndrome, male with 46, XX karyotype |
| Q98.3 | Other male with 46, XX karyotype |
| Q98.4 | Klinefelter's syndrome, unspecified |
| Q98.5 | Karyotype 47, XYY |
| Q98.6 | Male with structurally abnormal sex chromosome |
| Q98.7 | Male with sex chromosome mosaicism |
| Q98.8 | Other specified sex chromosome abnormalities, male phenotype |
| Q98.9 | Sex chromosome abnormality, male phenotype, unspecified |
| Q99 | Other chromosome abnormalities, NEC |
| Q99.0 | Chimera 46, XX/46, XY |
| Q99.0 | Chimera 46, XX/46, XY true hermaphrodite |
| Q99.1 | 46, XX true hermaphrodite |
| Q99.1 | 46,XX with streak gonads |
| Q99.1 | 46, Xy with streak gonads |
| Q99.1 | Pure gonadal dysgenesis |
| Q99.2 | Fragile X chromosome |
| Q99.2 | Fragile X syndrome |
| Q99.8 | Other specified chromosome abnormalities |
| Q99.9 | Chromosome abnormality, unspecified |

**Supplementary Table S2. ICD-10 codes used to specify birth weight of under 1,500g**

| **ICD-10 Code,  with subsections** | **Term** |
| --- | --- |
| P070.0 | Birth weight, less than 500 grams |
| P070.1 | Birth weight, 500 - 749 grams |
| P070.2 | Birth weight, 750 - 999 grams |
| P070.9 | Extremely low birth weight, unspecified weight |
| P071.0 | Birth weight, 1,000 – 1,249 grams |
| P071.1 | Birth weight, 1,250 – 1,499 grams |

**Supplementary Table S3. ICD-10 codes used to identify co-morbid diseases**

| **Co-morbidity** | **ICD-10 Code,  with subsections** | **Term** |
| --- | --- | --- |
| PDA ligation | O1671 | PDA ligaiton |
| Inraventricular hemorrhage | I61.5 | Intracerebral haemorrhage, intraventricular |
|  | P52.2 | Intraventricular (nontraumatic) haemorrhage, grade 3 and grade 4 of fetus and newborn |
| Pulmonary hypertension | P29.3 | Persistent pulmonary hypertension of newborn |
|  | I27.2 | Pulmonary hypertension |
|  | I27.0 | Pulmonary arterial hypertension |
| Necrotizing enterocolitis | P77 | Necrotizing enterocolitis of fetus and newborn |
| Retinopathy of prematurity | H35.1 | Retinopathy of prematurity |
| Sepsis | P36 | Bacterial sepsis of newborn, Congenital septicaemia |
|  | P36.0 | Sepsis of newborn due to streptococcus, group B |
|  | P36.1 | Sepsis of newborn due to other and unspecified streptococci |
|  | P36.2 | Sepsis of newborn due to Staphylococcus aureus |
|  | P36.3 | Sepsis of newborn due to other and unspecified staphylococci |
|  | P36.4 | Sepsis of newborn due to Escherichia coli |
|  | P36.5 | Sepsis of newborn due to anaerobes |
|  | P36.8 | Other bacterial sepsis of newborn |
|  | P36.9 | Bacterial sepsis of newborn, unspecified |
|  | A40 | Streptococcal sepsis |
|  | A40.0 | Sepsis due to streptococcus, group A |
|  | A40.1 | Sepsis due to streptococcus, group B |
|  | A40.2 | Sepsis due to streptococcus, group D |
|  | A40.3 | Sepsis due to Streptococcus pneumoniae, Pneumococcal sepsis |
|  | A40.8 | Other streptococcal sepsis |
|  | A40.9 | Streptococcal sepsis, unspecified |
|  | A41 | Other sepsis |
|  | A41.0 | Sepsis due to Staphylococcus aureus |
|  | A41.1 | Sepsis due to other specified staphylococcus, coagulase-negative staphylococcus |
|  | A41.2 | Sepsis due to unspecified staphylococcus |
|  | A41.3 | Sepsis due to Haemophilus influenzae |
|  | A41.4 | Sepsis due to anaerobes |
|  | A41.5 | Sepsis due to other Gram-negative organisms |
| **Co-morbidity** | **ICD-10 Code,  with subsections** | **Term** |
| Sepsis | A41.50 | Sepsis due to Escherichia coli[E. coli] |
|  | A41.52 | Sepsis due to Vibrio |
|  | A41.53 | Sepsis due to acinetobacter baumannii |
|  | A41.58 | Sepsis due to other Gram-negative organisms |
|  | A41.59 | Sepsis due to unspecified Gram-negative organisms |
|  | A41.8 | Other specified sepsis |
|  | A41.80 | Sepsis due to Enterococcus |
|  | A41.88 | Other specified sepsis |
|  | A41.9 | Sepsis, unspecified, septicaemia |

PDA, patent ductus arteriosus

**Supplementary Table S4. ICD-10 codes used to identify lower respiratory diseases**

| **Lower Respiratory Disease** | **ICD-10 Code,  with subsections** | **Term** |
| --- | --- | --- |
| Bronchitis | J45.9 | Asthma, unspecified |
|  | J45.9 | Asthmatic bronchitis NOS |
|  | J45.9 | Late-onset asthma |
|  | J40 | Bronchitis, not specified as acute or chronic |
|  | J40 | Bronchitis NOS |
|  | J40 | Catarrhal bronchitis |
|  | J40 | Bronchitis with tracheitis NOS |
|  | J40 | Tracheobronchitis NOS |
|  | J42 | Unspecified chronic bronchitis |
|  | J42 | Chronic bronchitis NOS |
|  | J42 | Chronic tracheitis |
|  | J42 | Chronic tracheobronchitis |
|  | J41.1 | Mucopurulent chronic bronchitis |
|  | J41.0 | Simple chronic bronchitis |
|  | J20.3 | Acute bronchitis due to coxsackievirus |
|  | J20.1 | Acute bronchitis due to Haemophilus influenzae |
|  | J20.7 | Acute bronchitis due to echovirus |
|  | J20.0 | Acute bronchitis due to Mycoplasma pneumoniae |
|  | J20.4 | Acute bronchitis due to parainfluenza virus |
|  | J20.5 | Acute bronchitis due to respiratory syncytial virus |
|  | J20.6 | Acute bronchitis due to rhinovirus |
|  | J20.9 | Acute bronchitis, unspecified |
| Bronchiolitis | J21.0 | Acute bronchiolitis due to respiratory syncytial virus |
|  | J21.9 | Acute bronchiolitis, unspecified |
|  | J84.8 | Other specified interstitial pulmonary diseases |
| Pneumonia | J15.9 | Bacterial pneumonia, unspecified |
|  | J18.9 | Pneumonia, unspecified |
|  | J18.0 | Bronchopneumonia, unspecified |
|  | B37.1 | Pulmonary candidiasis |
|  | J12.80 | Human bocavirus pneumonia |
|  | J11.0 | Influenza with pneumonia, virus not identified |
|  | J11.0 | Influenzal (broncho) pneumonia, unspecified or specific virus not identified |
|  | J10.0 | Influenza with pneumonia, seasonal influenza virus identified |
|  | J10.0 | Influenzal (broncho) pneumonia, other influenza virus identified |
|  | J84.9 | Interstitial pulmonary disease, unspecified |
|  | J84.9 | Interstitial pneumonia NOS |
|  | J15.0 | Pneumonia due to Klebsiella pneumoniae |
|  | J18.1 | Lobar pneumonia, unspecified |
|  | J15.7 | Pneumonia due to Mycoplasma pneumoniae |
|  | J70.9 | Respiratory conditions due to unspecified external agent |
| **Lower Respiratory Disease** | **ICD-10 Code,  with subsections** | **Term** |
| Pneumonia | J13 | Pneumonia due to Streptococcus pneumoniae |
|  | J13 | Bronchopneumonia due to S. pneumoniae |
|  | J15.5 | Pneumonia due to Escherichia coli |
|  | J15.6 | Pneumonia due to other aerobic Gram- negative bacteria |
|  | J15.6 | Pneumonia due to gram-negative (aerobic) bacteria NOS |
|  | J15.6 | Pneumonia due to serratia marcescens |
|  | J12.0 | Adenoviral pneumonia |
|  | J14 | Pneumonia due to Haemophilus influenzae |
|  | J14 | Bronchopneumonia due to H. influenzae |
|  | B05.2 | Measles complicated by pneumonia(J17.1*) |
|  | B05.2 | Postmeasles pneumonia(J17.1*) |
|  | J17.8 | Pneumonia in other diseases classified elsewhere |
|  | J17.8 | Pneumonia in ornithosis (A70+) |
|  | J17.8 | Pneumonia in Q fever (A78+) |
|  | J17.8 | Pneumonia in rheumatic fever (I00+) |
|  | J17.8 | Pneumonia in spirochaetal NEC (A69.8+) |
|  | J15.8 | Other bacterial pneumonia |
|  | J16.8 | Pneumonia due to other specified infectious organisms |
|  | J17.2 | Pneumonia in mycoses |
|  | J17.2 | Pneumonia in aspergillosis(B44.0-B44.1+) |
|  | J17.2 | Pneumonia in candidiasis(B37.1+) |
|  | J17.2 | Pneumonia in coccidioidomycosis(B38.0-B38.2+) |
|  | J17.2 | Pneumonia in histoplasmosis(B39.-+) |
|  | J12.2 | Parainfluenza virus pneumonia |
|  | B59 | Pneumocystosis(J17.3*) |
|  | B59 | Pneumonia due to Pneumocystis carinii(J17.3*) |
|  | B59 | Pneumonia due to Pneumocystis jirovecii(J17.3*) |
|  | B59 | Interstitial plasma cell pneumonia(J17.3*) |
|  | J15.1 | Pneumonia due to Pseudomonas |
|  | J12.1 | Respiratory syncytial virus pneumonia |
|  | J12.88 | Other viral pneumonia |
|  | J17.0 | Pneumonia in bacterial diseases classified elsewhere |
|  | J17.0 | Pneumonia due (to)(in) actinomycosis(A42.0+) |
|  | J17.0 | Pneumonia due (to)(in) anthrax(A22.1+) |
|  | J17.0 | Pneumonia due (to)(in) gonorrhoea(A54.8+) |
|  | J17.0 | Pneumonia due (to)(in) nocardiosis(A43.0+) |
|  | J17.0 | Pneumonia due (to)(in) salmonella infection(A02.2+) |
|  | J17.0 | Pneumonia due (to)(in) tularaemia(A21.2+) |
|  | J17.0 | Pneumonia due (to)(in) typhoid fever(A01.0+) |
|  | J17.0 | Pneumonia due (to)(in) whooping cough(A37.-+) |
|  | J15.2 | Pneumonia due to staphylococcus |
|  | J15.4 | Pneumonia due to other streptococci |
|  | J12.9 | Viral pneumonia, unspecified |
| Croup | J05.0 | Acute obstructive laryngitis [croup] |
|  | J05.0 | Obstructive laryngitis NOS |
| **Lower Respiratory Disease** | **ICD-10 Code,  with subsections** | **Term** |
| Laryngitis | J10.1 | Seasonal influenza virus identified influenza with other respiratory manifestations |
|  | J10.1 | Seasonal influenza virus identified influenza |
|  | J10.1 | Seasonal influenza virus identified influenzal acute upper respiratory infection |
|  | J10.1 | Seasonal influenza virus identified influenzal laryngitis |
|  | J10.1 | Seasonal influenza virus identified influenzal pharyngitis |
|  | J10.1 | Seasonal influenza virus identified influenzal pleural effusion |
|  | J37.1 | Chronic laryngotracheitis |
|  | J37.1 | Laryngitis, chronic, with tracheitis (chronic) |
|  | J37.1 | Tracheitis, chronic, with laryngitis |
|  | J04.0 | Acute laryngitis |
|  | J04.0 | Laryngitis(acute) NOS |
|  | J04.0 | Edematous laryngitis(acute) |
|  | J04.0 | Subglottic laryngitis(acute) |
|  | J04.0 | Supprative laryngitis(acute) |
|  | J04.0 | Ulcerative laryngitis(acute) |
|  | J06.0 | Acute laryngopharyngitis |
| Tracheitis | J37.1 | Chronic laryngotracheitis |
|  | J37.1 | Laryngitis, chronic, with tracheitis (chronic) |
|  | J37.1 | Tracheitis, chronic, with laryngitis |
|  | J04.2 | Acute laryngotracheitis |
|  | J04.2 | Laryngotracheitis NOS |
|  | J04.2 | Tracheitis (acute) with laryngitis (acute) |
|  | J04.1 | Acute tracheitis |
|  | J04.1 | Tracheitis (acute) NOS |
|  | J04.1 | Catarrhal tracheitis (acute) |

**Supplemenatary Table S5. Treatment codes used to identify ventilator use, non-invasive ventilator use, and oxygen prescription during hospital admissions**

| **Treatment** | **Treatment Code with subsections** |
| --- | --- |
| Ventilator use | M0850,M0850002,M0850003,M0850005,M0850010,M0850012,M0850013,M0850015,0850050,M0850052,M0850053,M0850055,M0850600,M0850610,M0850650,M0850A00,M0850A10,M0850A50,M0850B00,M0850B10,M0850B50,M0857,M0857002,M0857003,M0857005,M0857010,M0857012,M0857013,M0857015,M0857050,M0857052,M0857053,M0857055,M0857600,M0857610,M0857650,M0857A00,M0857A10,M0857A50,M0857B00,,M0857B10,M0857B50,M0858,M0858002,M0858003,M0858005,M0858010,M0858012,M0858013,M0858015,M0858050,M0858052,M0858053,M0858055,M0858600,M0858610,M0858650,M0858A00,M0858A10,M0858A50,M0858B00,M0858B10,M0858B50,M5850,,M5850002,M5850003,M5850005,M5850010,M5850012,M5850013,M5850015,M5850020,M5850022,M5850023,M5850025,M5850030,M5850032,M5850033,M5850035,M5850040,M5850042,M5850043,M5850045,M5850050,M5850052,M5850053,M5850055,M5850600,M5850610,M5850620,M5850630,M5850640,M5850650,M5850700,M5850710,M5850720,M5850750,M5850A00,M5850A10,M5850A20,M5850A30,M5850A40,M5850A50,M5850B00,M5850B10,M5850B20,M5850B30,M5850B40,M5850B50,M5857,M5857002,M5857003,M5857005,M5857010,M5857012,M5857013,M5857015,M5857020,M5857022,M5857023,M5857025,M5857030,M5857032,M5857033,M5857035,M5857040,M5857042,M5857043,M5857045,M5857050,M5857052,M5857053,M5857055,M5857600,M5857610,M5857620,M5857630,M5857640,M5857650,M5857700,M5857710,M5857720,M5857750,M5857A00,M5857A10,M5857A20,M5857A30,M5857A40,M5857A50,M5857B00,M5857B10,M5857B20,M5857B30,M5857B40,M5857B50,M5858,M5858002,M5858003,M5858005,M5858010,M5858012,M5858013,M5858015,M5858020,M5858022,M5858023,M5858025,M5858030,M5858032,M5858033,M5858035,M5858040,M5858042,M5858043,M5858045,M5858050,M5858052,M5858053,M5858055,M5858600,M5858610,M5858620,M5858630,M5858640,M5858650,M5858700,M5858710,M5858720,M5858750,M5858A00,M5858A10,M5858A20,M5858A30,M5858A40,M5858A50,M5858B00,M5858B10,M5858B20,M5858B30,M5858B40,M5858B50,M5859,M5859002,M5859003,M5859005,M5859010,M5859012,M5859013,M5859015,M5859020,M5859022,M5859023,M5859025,M5859030,M5859032,M5859033,M5859035,M5859040,M5859042,M5859043,M5859045,M5859050,M5859052,M5859053,M5859055,M5859600,M5859610,M5859620,M5859630,M5859640,M5859650,M5859700,M5859710,M5859720,M5859750,M5859A00, |
| **Treatment** | **Treatment Code with subsections** |
| Ventilator use | M5859A10,M5859A20,M5859A30,M5859A40,M5859A50,M5859B00,M5859B10,M5859B20,M5859B30,M5859B40,M5859B50,M5860,M5860002,M5860003,M5860005,M5860010,M5860012,M5860013,M5860015,,M5860020,M5860022,M5860023,M5860025,M5860030,M5860032,M5860033,M5860035,M5860040,M5860042,M5860043,M5860045,M5860050,M5860052,M5860053,M5860055,M5860600,M5860610,M5860620,M5860630,M5860640,M5860650,M5860A00,M5860A10,M5860A20,M5860A30,M5860A40,M5860A50,M5860B00,M5860B10,M5860B20,M5860B30,M5860B40,M5860B50 |
| Noninvasive ventilator use | MM360,MM400,M0046,M0046002,M0046003,M0046005,M0046010,M0046012,M0046013,M0046015,M0046050,M0046052,M0046053,M0046055,M0046600,M0046610,M0046650,M0046A00,M0046A10,M0046A50,M0046B00,M0046B10,M0046B50 |
| Oxygen use | M0040,M0040002,M0040003,M0040005,M0040010,M0040012,M0040013,M0040015,M0040020,M0040022,M0040023,M0040025,M0040030,M0040032,M0040033,M0040035,M0040040,M0040042,M0040043,M0040045,M0040050,M0040052,M0040053,M0040055,M0040090,M0040092,M0040093,M0040095,M00400A0,M00400A2,M00400A3,M00400A5,M00400B0,M00400B2,M00400B3,M00400B5,M0040600,M0040610,M0040620,M0040630,M0040640,M0040650,M0040A00,M0040A10,M0040A20,M0040A30,,M0040A40,M0040A50,M0040B00,M0040B10,M0040B20,M0040B30,M0040B40,M0040B50 |

**Supplementary Table S6. Classification of cause of death using ICD-10 codes**

| **Classification** | **ICD-10 Code  for cause of death** | **Term** |
| --- | --- | --- |
| Respiratory Disease | P22 | Respiratory distress of newborn |
|  | P27 | Chronic respiratory disease originating in the perinatal period |
|  | J18 | Pneumonia, organism unspecified |
|  | P24 | Neonatal aspiration syndromes |
|  | Q33 | Congenital malformations of lung |
|  | J69 | Pneumonitis due to solids and liquids |
|  | J21 | Acute bronchiolitis |
|  | I27 | Other pulmonary heart diseases |
|  | P26 | Pulmonary haemorrhage originating in the perinatal period |
|  | P28 | Other respiratory conditions originating in the perinatal period |
|  | J20 | Acute bronchitis |
|  | J45 | Asthma |
|  | J96 | Respiratory failure, NEC |
|  | J98 | Other respiratory disorders |
|  | J04 | Acute laryngitis and tracheitis |
|  | J09 | Influenza due to identified zoonotic or pandemic influenza virus |
|  | J84 | Other interstitial pulmonary diseases |
|  | J93 | Pneumothorax |
|  | P23 | Congenital pneumonia |
|  | R06 | Abnormalities of breathing |
| Non-respiratory Disease | P77 | Necrotizing enterocolitis of fetus and newborn |
|  | P36 | Bacterial sepsis of newborn |
|  | G80 | Infantile cerebral palsy |
|  | A00-B99 | Certain infectious and parasitic diseases |
|  | P91 | Other disturbances of cerebral status of newborn |
|  | P29 | Cardiovascular disorders originating in the perinatal period |
|  | G93 | Other disorders of brain |
|  | P78 | Other perinatal digestive system disorders |
|  | P52 | Intracranial nontraumatic haemorrhage of fetus and newborn |
|  | G40 | Epilepsy |
|  | G91 | Hydrocephalus |
|  | P76 | Other intestinal obstruction of newborn |
|  | G41 | Status epilepticus |
|  | P21 | Birth asphyxia |
|  | Q04 | Other congenital malformations of brain |
|  | Q25 | Congenital malformations of great arteries |
|  | G71 | Primary disorders of muscles |
|  | G12 | Spinal muscular atrophy and related syndromes |
|  | P35 | Congenital viral diseases |
|  | C71 | Malignant neoplasm of brain |
|  | G00 | Bacterial meningitis, NEC |
|  | I40 | Acute myocarditis |
|  | I46 | Cardiac arrest |
| **Classification** | **ICD-10 Code  for cause of death** | **Term** |
| Non-respiratory Disease | N18 | Chronic renal failure |
|  | P11 | Other birth injuries to central nervous system |
|  | P60 | Disseminated intravascular coagulation of fetus and newborn |
|  | Q24 | Other congenital malformations of heart |
|  | R09 | Other symptoms and signs involving the circulatory and respiratory systems |
|  | C49 | Malignant neoplasm of other connective and soft tissue |
|  | C74 | Malignant neoplasm of adrenal gland |
|  | C92 | Myeloid leukaemia |
|  | E84 | Cystic fibrosis |
|  | E87 | Other disorders of fluid, electrolyte and acid-base balance |
|  | G04 | Encephalitis, myelitis and encephalomyelitis |
|  | G31 | Other degenerative diseases of nervous system NEC |
|  | I62 | Other nontraumatic intracranial haemorrhage |
|  | J12 | Viral pneumonia, NEC |
|  | P37 | Other congenital infectious and parasitic diseases |
|  | P96 | Other conditions originating in the perinatal period |
|  | Q03 | Congenital hydrocephalus |
|  | Q21 | Congenital malformations of cardiac septa |
|  | Q74 | Other congenital malformations of limb(s) |
|  | R57 | Shock, NEC |
|  | C22 | Malignant neoplasm of liver and intrahepatic bile ducts |
|  | C69 | Malignant neoplasm of eye and adnexa |
|  | C76 | Malignant neoplasm of other and ill-defined sites |
|  | C83 | Diffuse non- Hodgkin's lymphoma |
|  | C91 | Lymphoid leukaemia |
|  | C94 | Other leukaemias of specified cell type |
|  | C95 | Leukaemia of unspecified cell type |
|  | D18 | Haemangioma and lymphangioma, any site |
|  | D33 | Benign neoplasm of brain and other parts of central nervous system |
|  | D37 | Neoplasm of uncertain or unknown behaviour of oral cavity and digestive organs |
|  | D81 | Combined immunodeficiencies |
|  | E25 | Adrenogenital disorders |
|  | E26 | Hyperaldosteronism |
|  | E83 | Disorders of mineral metabolism |
|  | E86 | Volume depletion |
|  | G72 | Other myopathies |
|  | G95 | Other diseases of spinal cord |
|  | I31 | Other diseases of pericardium |
|  | I33 | Acute and subacute endocarditis |
|  | I42 | Cardiomyopathy |
|  | I51 | Complications and ill-defined descriptions of heart disease |
|  | I61 | Intracerebral haemorrhage |
|  | I69 | Sequelae of cerebrovascular disease |
|  | I71 | Aortic aneurysm and dissection |
| **Classification** | **ICD-10 Code  for cause of death** | **Term** |
| Non-respiratory Disease | I89 | Other noninfective disorders of lymphatic vessels and lymph nodes |
|  | K55 | Vascular disorders of intestine |
|  | K59 | Other functional intestinal disorders |
|  | K63 | Other diseases of intestine |
|  | K72 | Hepatic failure, NEC |
|  | K74 | Fibrosis and cirrhosis of liver |
|  | K92 | Other diseases of digestive system |
|  | N04 | Nephrotic syndrome |
|  | N17 | Acute renal failure |
|  | N19 | Unspecified renal failure |
|  | P54 | Other neonatal haemorrhages |
|  | P61 | Other perinatal haematological disorders |
|  | P94 | Disorders of muscle tone of newborn |
|  | Q02 | Microcephaly |
|  | Q28 | Other congenital malformations of circulatory system |
|  | Q43 | Other congenital malformations of intestine |
|  | Q44 | Congenital malformations of gallbladder, bile ducts and liver |
| External Causes | S00-T98 | Injury, poisoning, and certain other consequences of external causes |
| Miscellaneous | R95 | Sudden infant death syndrome |
|  | P07 | Disorders related to short gestation and low birth weight, NEC |
|  | R99 | Other ill-defined and unspecified causes of mortality |
|  | R96 | Other sudden death, cause unknown |

**Supplementary Table S7. Active ingredient codes used to identify asthma medications prescribed for asthma patients**

| **Active ingredient code** | **Pharmaceutical drug** |
| --- | --- |
| 114508CSI | beclomethasone dipropionate 12mg |
| 114509CSI | beclomethasone dipropionate 20mg |
| 114510CSI | beclomethasone dipropionate 6mg |
| 119403CAE | budesonide 24mg |
| 119404CSI | budesonide 500㎍ |
| 119407CAE | budesonide 40mg |
| 119438CAE | budesonide 40mg(0.2mg/administration) |
| 119502CSI | budesonide(micronized) 20mg |
| 119505CSI | budesonide(micronized) 40mg |
| 119506CSI | budesonide(micronized) 80mg |
| 119530CSI | budesonide(micronized) 20mg(0.2mg/administration) |
| 119531CSI | budesonide(micronized) 40mg(0.2mg/administration) |
| 119533CSI | budesonide(micronized) 0.5mg(0.25mg/mL) |
| 162202CSI | fluticasone propionate 15mg |
| 162203CSS | fluticasone propionate 2mg |
| 162204CSI | fluticasone propionate 30mg |
| 162205CSI | fluticasone propionate 6mg |
| 162206CSS | fluticasone propionate 0.5mg |
| 162203CLQ | fluticasone propionate 2mg |
| 162230CSS | fluticasone propionate 0.5mg(0.25mg/mL) |
| 162231CSS | fluticasone propionate 2mg(1mg/mL) |
| 162232CSI | fluticasone propionate 6mg(50μg/administration) |
| 162233CSI | fluticasone propionate 6mg(0.1mg/administration) |
| 162235CSI | fluticasone propionate 15mg(0.25mg/administration) |
| 162236CSI | fluticasone propionate 30mg(0.25mg/administration) |
| 497101CSI | ciclesonide 4.8mg |
| 497102CSI | ciclesonide 9.6mg |
| 497130CSI | ciclesonide 4.8mg(80μg/administration) |
| 497131CSI | ciclesonide 9.6mg(0.16mg/administration) |
| 542800CSI | fluticasone propionate 15mg(0.125mg/administration) |
| 542900CSI | fluticasone propionate 6mg(50μg/administration) |
| 543000CSI | fluticasone propionate 30mg(0.25mg/administration) |
| 334500CSI | fluticasone propionate 6mg |
| 334600CSI | fluticasone propionate 15mg |
| 334700CSI | fluticasone propionate 30mg |
| 391800CSI | (micronized)budesonide 180mg/g |
| 407100CSI | fluticasone propionate 20mg |
| 407200CSI | fluticasone propionate 40mg |
| 407300CSI | fluticasone propionate 8mg |
| 441700COS | (micronized)budesonide 90mg/g |
| 441700CSI | (micronized)budesonide 90mg/g |
| 453400CSI | (micronized)budesonide 390mg/g |
| 502000CSI | beclomethasone dipropionate 172.41mg |
| 506400CSI | fluticasone propionate 2.8mg |
| 506500CSI | fluticasone propionate 7mg |
| 506600CSI | fluticasone propionate 14mg |
| 525700CSI | fluticasone propionate 15mg |
| 525800CSI | fluticasone propionate 6mg |
| 526200CSI | fluticasone propionate 30mg |
| 543100CSI | fluticasone propionate 6mg(0.1mg/administration) |
| 543200CSI | fluticasone propionate 6mg(50μg/administration) |
| 543300CSI | fluticasone propionate 15mg(0.25mg/administration) |
| 543400CSI | fluticasone propionate 15mg(0.125mg/administration) |
| 543500CSI | fluticasone propionate 30mg(0.5mg/administration) |
| 543600CSI | fluticasone propionate 30mg(0.25mg/administration) |
| 543800CSI | budesonide(micronized) 9.6mg(0.16mg/administration) |
| 543900CSI | budesonide(micronized) 19.2mg(0.16mg/administration) |
| 544000CSI | budesonide(micronized) 4.8mg(80μg/administration) |
| 544100CSI | budesonide(micronized) 19.2mg(0.32mg/administration) |
| 544200CSI | beclomethasone dipropionate 12mg(0.1mg/administration) |
| 544300CSI | fluticasone propionate 2.8mg(0.1mg/administration) |
| 544400CSI | fluticasone propionate 7mg(0.25mg/administration) |
| 544500CSI | fluticasone propionate 14mg(0.5mg/administration) |
| 636700CSI | fluticasone furoate(micronized) 3mg(100μg/administration) |
| 636800CSI | fluticasone furoate(micronized) 6mg(200μg/administration) |
| 801100CSI | budesonide(micronized) 9.6mg(80μg/administration) |
| 216401ACH | pranlukast hydrate 0.1125g |
| 216402ASS | pranlukast hydrate 0.1g |
| 216403ACH | pranlukast hydrate 0.1125g/administration |
| 216404ATB | pranlukast hydrate 75mg |
| 216405ASS | pranlukast hydrate 50mg |
| 216405ATB | pranlukast hydrate 50mg |
| 216406ASS | pranlukast hydrate 70mg |
| 216407ASS | pranlukast hydrate 0.14g |
| 216408ATB | pranlukast hydrate 50mg |
| 216430ASY | pranlukast hydrate 50mg(0.1g/g) |
| 216431ASY | pranlukast hydrate 70mg(0.1g/g) |
| 216432ASY | pranlukast hydrate 0.1g(0.1g/g) |
| 216433ASY | pranlukast hydrate 10g(0.1g/g) |
| 249701ATB | zafirlukast 20mg |
| 374601ASY | montelukast sodium (as montelukast 5mg) |
| 374601ATB | montelukast sodium (as montelukast 5mg) |
| 374601ATD | montelukast sodium (as montelukast 5mg) |
| 374602ATB | montelukast sodium (as montelukast 10mg) |
| 374602ATD | montelukast sodium (as montelukast 10mg) |
| 374603AGN | montelukast sodium (as montelukast 4mg) |
| 374603ASY | montelukast sodium (as montelukast 4mg) |
| 374603ATB | montelukast sodium (as montelukast 4mg) |
| 374603ATD | montelukast sodium (as montelukast 4mg) |
| 659900ACH | montelukast sodium (as montelukast 10mg) |
| 116401ATB | betamethasone 0.5mg |
| 140801ATB | deflazacort 6mg |
| 141901ATB | dexamethasone 0.5mg |
| 141903ATB | dexamethasone 0.75mg |
| 141904ATB | dexamethasone 4mg |
| 170901ATB | hydrocortisone 10mg |
| 170905ATB | hydrocortisone 20mg |
| 170906ATB | hydrocortisone 5mg |
| 193302ATB | methylprednisolone 4mg |
| 193304ATB | methylprednisolone aceponate 2mg |
| 193305ATB | methylprednisolone 1mg |
| 217001ATB | prednisolone 5mg |
| 217003ASY | prednisolone 1mg |
| 217004ASY | prednisolone 3mg |
| 217030ASY | prednisolone 3mg(3mg/mL) |
| 217034ASY | prednisolone 0.5g(1mg/mL) |
| 217035ASY | prednisolone 1.5g(3mg/mL) |
| 243201ATB | triamcinolone 1mg |
| 243202ATB | triamcinolone 2mg |
| 243203ATB | triamcinolone 4mg |
| 296900ATB | betamethasone 0.25mg |
| 116502BIJ | betamethasone sodium phosphate 4mg |
| 116530BIJ | betamethasone sodium phosphate (as betamethasone 4mg(4mg/mL)) |
| 142030BIJ | dexamethasone palmitate 4mg(4mg/mL) |
| 142230BIJ | dexamethasone disodium phosphate 4.37mg(4.37mg/mL) |
| 142232BIJ | dexamethasone disodium phosphate 5mg(5mg/mL) |
| 142201BIJ | dexamethasone sodium phosphate 5mg |
| 142202BIJ | dexamethasone sodium phosphate 4.37mg |
| 142233BIJ | dexamethasone disodium phosphate 20mg(5mg/mL) |
| 171201BIJ | hydrocortisone sodium succinate (as hydrocortisone 0.1g) |
| 171202BIJ | hydrocortisone sodium succinate (as hydrocortisone 0.25g) |
| 193530BIJ | methylprednisolone acetate 40mg(40mg/mL) |
| 193531BIJ | methylprednisolone acetate 0.2g(40mg/mL) |
| 193601BIJ | methylprednisolone sodium succinate (as methylprednisolone 0.125g) |
| 193501BIJ | methylprednisolone acetate 200mg |
| 193502BIJ | methylprednisolone acetate 40mg |
| 193602BIJ | methylprednisolone sodium succinate (as methylprednisolone 0.25g) |
| 193603BIJ | methylprednisolone sodium succinate (as methylprednisolone 40mg) |
| 193604BIJ | methylprednisolone sodium succinate (as methylprednisolone 0.5g) |
| 217302BIJ | prednisolone sodium succinate 0.25g |
| 243301BIJ | triamcinolone acetonide 200mg |
| 243303BIJ | triamcinolone acetonide 40mg |
| 243305BIJ | triamcinolone acetonide 50mg |
| 243335BIJ | triamcinolone acetonide 40mg(40mg/mL) |
| 243336BIJ | triamcinolone acetonide 50mg(10mg/mL) |
| 243337BIJ | triamcinolone acetonide 0.2g(40mg/mL) |
| 107301ATB | aminophylline hydrate 0.1g |
| 107301ATR | aminophylline hydrate 0.1g |
| 107303ATR | aminophylline hydrate 0.225g |
| 236901ASY | theophiline anhydrous 5.333mg. |
| 237001ACH | theophylline 100mg |
| 237001ACR | theophylline 100mg |
| 237002ACR | theophylline 130mg |
| 237003ACH | theophylline 200mg |
| 237003ACR | theophylline 200mg |
| 237003ASY | theophylline 200mg |
| 237003ATR | theophylline 200mg |
| 237005ATR | theophylline 400mg |
| 107302BIJ | aminophylline 250mg |
| 107330BIJ | aminophylline 0.25g(25mg/mL) |
| 177131CLQ | ipratropium bromide monohydrate 0.5mg(0.25mg/mL) |
| 218330CSI | procaterol hydrochloride(micronized) 2mg(10μg/administration) |
| 225502CSI | salbutamol sulfate(as salbutamol) 20mg |
| 225530CSI | salbutamol sulfate (as salbutamol 2.5mg(1mg/mL)) |
| 225531CSI | salbutamol sulfate (as salbutamol 20mg(0.1mg/administration)) |
| 225532CSI | salbutamol sulfate (as salbutamol 0.1g(5mg/mL)) |
| 114530CSI | beclomethasone dipropionate 6mg(0.1mg/administration) |
| 114532CSI | beclomethasone dipropionate 12mg(0.1mg/administration) |
| 114533CSI | beclomethasone dipropionate 20mg(0.1mg/administration) |
| 157902CLQ | fenoterol Hbr 500㎍ |
| 177104CAE | ipratropium bromide 6mg |
| 177101CLQ | ipratropium bromide 261㎍ |
| 177103CLQ | ipratropium bromide 521.8㎍ |
| 225802CSI | salmeterol 3mg |
| 225504CSI | salmeterol |
| 225506CSI | salbutamol sulfate 3mg |
| 225508CSI | salbutamol sulfate 6mg |
| 235805CLQ | terbutaline sulfate 5mg |
| 334800CAE | ipratropium bromide 4.2mg |
| 157901ATB | fenoterol Hbr 2.5mg |
| 163101ATB | formoterol fumarate 20㎍ |
| 163104ASY | formoterol fumarate 40㎍ |
| 163104ATB | formoterol fumarate 40㎍ |
| 218302ATB | procaterol HCl 50㎍ |
| 225501ATB | salbutamol sulfate(as salbutamol) 2mg |
| 225503ACR | salbutamol sulfate(as salbutamol) 4mg |
| 225503ATB | salbutamol sulfate(as salbutamol) 4mg |
| 225507ACR | salbutamol sulfate(as salbutamol) 8mg |
| 235801ATB | terbutaline sulfate 2mg |
| 267000ATB | aminophylline 50mg |
| 142001BIJ | dexamethasone palmitate 4mg |
| 457301CCH | micronized tiotropium bromide monohydrate(as tiotropium 18mcg) 22.5mcg |
| 457301CSI | micronized tiotropium bromide monohydrate(as tiotropium 18mcg)/cap 22.5mcg |
| 503401CSI | tiotropium bromide monohydrate 1272.15mcg/4.5ml |
| 611901CSI | indacaterol maleate(as indacaterol) 150mcg/cap |
| 611902CSI | indacaterol maleate(as indacaterol) 300mcg/cap |
| 627500CSI | indacaterol maleate(as indacaterol 110mcg) 0.143mg/capsule |
| 631200CSI | micronized vilanterol trifenatate (as vilanterol 750㎍ (25㎍/administration)) |
| 633701CSI | micronized aclidinium bromide 24mg (400㎍/administration) |
| 635300CSI | micronized aclidinium bromide 24mg (400μg/administration) |
| 635300CSI | formoterol fumarate dihydrate 0.72mg (12μg/administration) |
| 119501CMS | budesonide(micronized) 2.3mg |
| 267100ACH | aminophylline 50mg |
| 114511ATE | beclomethasone dipropionate 5mg |
| 113801ATB | bamiphylline HCl 300mg |
| 113802ATB | bamiphylline HCl 600mg |
| 135301ASY | clenbuterol HCl 1㎍ |
| 264700ASY | clenbuterol HCl 1㎍ |
| 264800ATB | clenbuterol HCl 20㎍ |
| 439101ATB | doxofylline 400mg |
| 113601ATB | bambuterol HCl 10mg |
| 113602ASY | bambuterol HCl 1mg |
| 495700CSI | ipratropium bromide 6mg |
| 640400CSI | (micronized)budesonide 80㎍/administration |
| 640400CSI | formoterol fumartate. 2H2O 2.25㎍/administration |
| 163130ASY | formoterol fumarate hydrate 4mg(40μg/g) |
| 163131ASY | formoterol fumarate hydrate 12mg(40μg/g) |
| 218301ATB | procaterol hydrochloride 25μg |
| 237030ASY | theophylline 0.2g(0.2g/g) |
| 237031ASY | theophylline 40g(0.2g/g) |
| 531700ASY | clenbuterol hydrochloride 60μg(1μg/mL) |
| 531800ASY | clenbuterol hydrochloride 0.1mg(1μg/mL) |
| 531900ASY | clenbuterol hydrochloride 0.45mg(1μg/mL) |
| 532000ASY | clenbuterol hydrochloride 0.5mg(1μg/mL) |
| 532100ASY | clenbuterol hydrochloride 1mg(1μg/mL) |
| 532200ASY | clenbuterol hydrochloride 2mg(1μg/mL) |
| 532300ASY | clenbuterol hydrochloride 4mg(1μg/mL) |
| 113630ASY | bambuterol hydrochloride 0.5g(1mg/mL) |
| 119532CSI | budesonide(micronized) 80mg(0.4mg/administration) |
| 235830CLQ | terbutaline sulfate 5mg(2.5mg/mL) |
| 157930CLQ | fenoterol hydrobromide 0.5mg(0.25mg/mL) |
| 457330CSI | tiotropium bromide monohydrate(micronized) (as tiotropium 0.54mg(18μg/capsule)) |
| 503430CSI | tiotropium bromide monohydrate 0.3mg(5μg/administration) |
| 800100CSI | indacaterol maleate (as indacaterol 3.3mg(0.11mg/capsule)) |
| 135330ASY | clenbuterol hydrochloride 60μg(1μg/mL) |
| 135331ASY | clenbuterol hydrochloride 1mg(1μg/mL) |

**Supplementary Table S8. Cause of death among patients who died within 2 years of birth**

|  | **BPD** | **Non-BPD** | ***P*-value** |
| --- | --- | --- | --- |
|  | **(n = 264)** | **(n = 387)** |  |
| Respiratory morbidity | 112 (42.4) | 98 (25.3) | <0.001 |
| Non-respiratory morbidity | 118 (44.7) | 197 (50.9) | 0.14 |
| Accident | 9 (3.4) | 36 (9.3) | 0.006 |
| Unknown cause | 15 (5.7) | 44 (11.4) | 0.019 |
| Age at death, years | 0.52 (0.28–1.01) | 0.38 (0.14–1.22) | 0.003 |

Data expressed as n (%) or median (interquartile range). BPD, bronchopulmonary dysplasia

**Supplementary Table S9. Readmission due to lower respiratory disease in 2 years (2011-2015)**

|  | BPD (n = 5,907) | Non-BPD (n = 27,530) | *P*- value | <1,500g  (n = 6,774) | >1,500g  (n = 26,663) | *P*- value | <1,500g | | | >1,500g | | |
| --- | --- | --- | --- | --- | --- | --- | --- | --- | --- | --- | --- | --- |
|  |  |  |  |  |  |  | BPD  (n = 3,981) | Non-BPD  (n = 2,793) | *P*- value | BPD  (n = 1,926) | Non-BPD  (n = 24,737) | *P*- value |
| Readmission | 3,136  (53.1) | 10,765  (39.1) | <0.001 | 3,430  (50.6) | 10,471  (39.3) | <0.001 | 2,184  (54.9) | 1,246  (44.6) | <0.001 | 952  (49.4) | 9,519  (38.5) | <0.001 |
| Number of readmissions* | 2 (1-3) | 1  (1-3) | <0.001 | 2  (1-3) | 1  (1-3) | <0.001 | 2  (1-3) | 2  (1-3) | <0.001 | 2  (1-3) | 1  (1-3) | <0.001 |
| Hospital days | 12  (6-28) | 8  (5-15) | <0.001 | 11  (6-25) | 8  (5-15) | <0.001 | 13  (6-31) | 9  (5-18) | <0.001 | 11  (5-22) | 8  (5-15) | <0.001 |
| Ventilator use | 443  (7.5) | 245  (0.9) | <0.001 | 411  (12.0) | 277  (2.6) | <0.001 | 347  (15.9) | 64  (5.1) | <0.001 | 96  (10.1) | 181  (1.9) | <0.001 |
| NIV use | 185  (3.1) | 80  (0.3) | <0.001 | 175  (5.1) | 90  (0.9) | <0.001 | 151  (6.9) | 24  (1.9) | <0.001 | 34  (3.6) | 56  (0.6) | <0.001 |
| Oxygen use | 1,240  (21.0) | 1,305  (4.7) | <0.001 | 1,164  (33.9) | 1,381  (13.2) | <0.001 | 936  (42.9) | 228  (18.3) | <0.001 | 304  (31.9) | 1,077  (11.3) | <0.001 |
| ICU admission | 598  (10.1) | 435  (1.6) | <0.001 | 578  (8.5) | 455  (1.7) | <0.001 | 468  (11.8) | 110  (3.9) | <0.001 | 130  (6.7) | 325  (1.3) | <0.001 |
| Number of ICU admissions* | 1 (1-4) | 1  (1-8) | <0.001 | 1  (1-4) | 1  (1-8) | <0.001 | 1  (1-4) | 1  (1-2) | 0.028 | 1  (1-4) | 1  (1-8) | 0.065 |
| ICU hospital days | 38  (18-64) | 14  (8-27) | <0.001 | 38  (17-63) | 15  (9-30) | <0.001 | 42  (21-68) | 23  (10-49) | <0.001 | 30  (14-55) | 13  (8-22) | <0.001 |

Data are expressed as n (%), median (interquartile range), or median (range)*. The Mann–Whitney U test was used for non-normal distributions in continuous variables. BPD, bronchopulmonary dysplasia; NIV, non-invasive ventilator; ICU, intensive care unit

**Supplementary Figure S1. Patient flow diagram and analysis subjects**

**
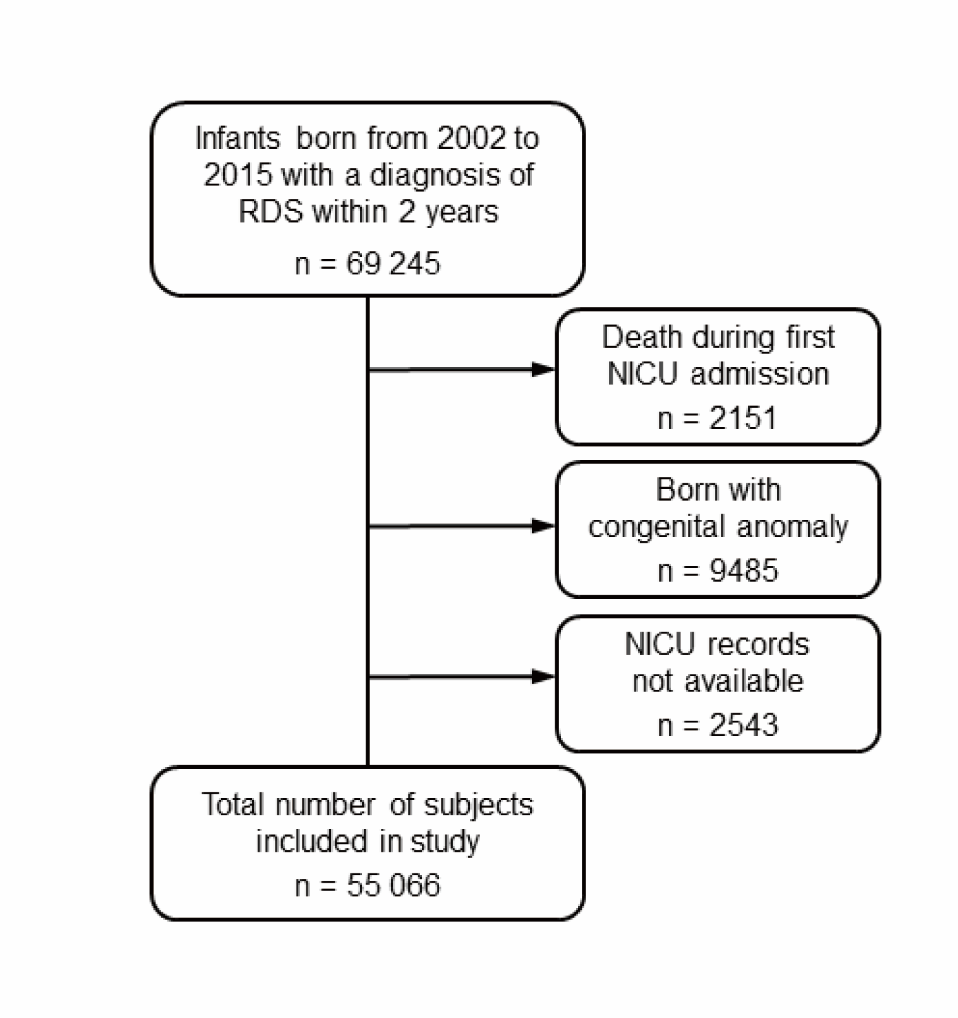
**

Patient flow diagram and analysis subjects. RDS, respiratory distress syndrome.

**Supplementary Figure S2. Relative risk of BPD on readmission due to respiratory diseases**


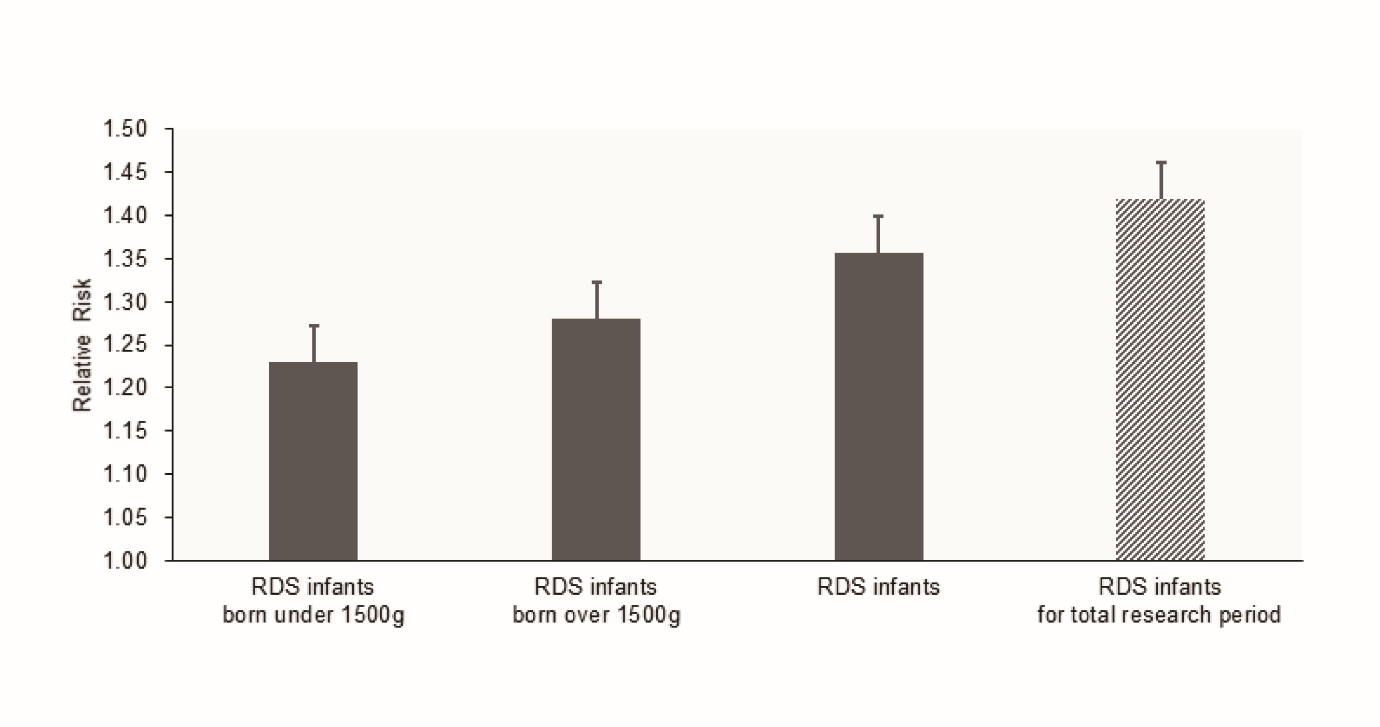


Relative risk of BPD combined with birth weight category on readmission due to respiratory disease. BPD, bronchopulmonary dysplasia; RDS, respiratory distress syndrome.
